# The *Porphyromonas gingivalis* RNA-binding protein is required for growth in high levels of zinc and persistence with host cells

**DOI:** 10.3389/fcimb.2025.1569544

**Published:** 2025-05-15

**Authors:** Sai Yanamandra, Holly Marsh, Romana Cvitkovic, Qin Gui, Benjamin R. Belvin, Janina P. Lewis

**Affiliations:** ^1^ Philips Institute for Oral Health Research, Virginia Commonwealth University, Richmond, VA, United States; ^2^ Department of Microbiology and Immunology, Virginia Commonwealth University, Richmond, VA, United States; ^3^ Department of Biochemistry, Virginia Commonwealth University, Richmond, VA, United States

**Keywords:** RNA-binding protein, RRM-1, *Porphyromonas gingivalis*, zinc homeostasis, protease activity, host–pathogen interaction, bacteroides

## Abstract

The oral periodontal pathogen *Porphyromonas gingivalis* must adapt to an ever-changing environment to survive and cause disease. So far, most of the efforts concerning the regulatory mechanisms employed by the bacterium centered on DNA-binding regulators. Although global regulatory mechanisms employing RNA-binding proteins (RBP) are reported in most forms of life so far, such mechanism of regulation remains unknown in the oral Bacteroidetes group. Examination of the genome of *P. gingivalis* led to the discovery of a putative RBP with the RNA recognition motif 1 (RRM-1) designated here RbpPg1 (RNA-binding protein *Porphyromonas gingivalis* 1). The recombinant form of the protein-bound RNA and RNA-pull down identified a zinc exporter transcript as the most enriched one in agreement with the higher levels of zinc in the absence of the protein. Deletion of RbpPg1 reduced the ability of the bacterium to grow with 0.5 mM zinc. The RgpB protein level and the Arg-X protease activity was reduced in both iron replete and iron deplete conditions in the mutant strain when compared to the wild type. Lys-X protease activity was reduced, although Kgp protein levels were not altered by deletion of RbpPg1. The mutant grew better in hemin-deplete conditions when compared to the wild type. Finally, RbpPg1 was indispensable for the bacterium to survive with host cells. We have determined that both the transcriptome and proteome are affected by the deletion of RbpPg1 and found that the major group of proteins with elevated expression were the ones associated with response to environmental stress changes, while proteins mediating metabolic processes were downregulated. Overall, the first RBP characterized in *P. gingivalis* plays a significant role in the biology of the bacterium and differs from RBPs in other Gram-negative bacteria. Data are available via ProteomeXchange with identifier PXD034144 and via the NCBI Gene Expression Omnibus (GEO) and under accession number GSE168570.

## Introduction

*Porphyromonas gingivalis*, a Gram-negative anaerobic bacterium, is implicated as a major etiological agent in adult-onset periodontal disease (Lamont and Jenkinson, 1998; Hajishengallis et al., 2012; Hajishengallis and Lamont, 2014). This disease is a chronic inflammatory condition of the supporting tissues of the teeth affecting 10%–15% of adults (Fox, 1992; Petersen and Ogawa, 2005). The annual economic burden associated with those diseases was estimated to be $14.3 billion in the USA alone; however, it may be higher than estimated due to multiple comorbidities associated with chronic periodontitis including increased risk of cardiovascular diseases (Beck et al., 1998; Miyakawa et al., 2004; Carra et al., 2023), diabetes (Pucher and Stewart, 2004; Guo et al., 2018; Ahmad and Haque, 2021), rheumatoid arthritis (Mercado et al., 2003; Potempa et al., 2017), and spontaneous preterm birth and preterm low birth weight (Champagne et al., 2000; Shira Davenport, 2010; Usin et al., 2016; Montoya-Carralero et al., 2024).

To reach the periodontal pockets, where *P. gingivalis* is commonly found, it is transferred through different sites in the oral cavity (saliva, tongue, buccal mucosa) where it is exposed to various conditions (presence of oxygen and nitrite, variation in nutrient availability) (Lamont et al., 1995; Rosier et al., 2018; Wade, 2021; Cato et al., 2023; du Toit et al., 2024). Additionally, it is present in oral biofilms where it is exposed to various factors such as peroxide produced by other members of the community, especially *Streptococci (* Auzat et al., 1999; Barnard and Stinson, 1999; Barnard and Stinson, 1999; Lewin et al., 2024). P*. gingivalis* has been demonstrated to invade and survive within eukaryotic cells where it encounters intracellular oxidative and nitrosative stress and altered nutrient availability different from that in the extracellular environment (Papapanou et al., 1994; Rudney et al., 2001; Mydel et al., 2006; Li et al., 2008; Henry et al., 2014; Sakanaka et al., 2016). Last, *P. gingivalis* must contend with the host immune response that produces reactive oxygen, nitrogen, and sulfur species that are generated by macrophages and neutrophils upon encounter with bacterial cells (Chapple, 1997; Zheng et al., 2021; Maquera-Huacho et al., 2022). Thus, to adapt to and survive the various conditions in the human host, *P. gingivalis* has had to develop efficient adaptation mechanisms that would allow it to adjust its protein expression accordingly.

Most, if not all, bacteria contain numerous small non-coding RNA species (sRNA) that play a crucial role as regulatory elements in bacterial stress responses and virulence (Massé et al., 2003; Majdalani et al., 2005; Gottesman et al., 2006; Gripenland et al., 2010; Storz et al., 2011; Oliva et al., 2015). sRNAs can modulate gene expression post-transcriptionally by altering the translation or stability of mRNAs. These RNAs are complementary to mRNA targets in the 5′ or 3′ untranslated region and can either inhibit translation by occluding the Shine Dalgarno (SD) sequences or stimulate translation by revealing SD sequences for translation. Also, hybridization with 3′UTR sequences can stabilize mRNA as well. Although the presence of multiple sRNAs in *P. gingivalis* has been shown, the targets and mechanism of regulation by the sRNAs is yet to be investigated (Hirano et al., 2012; Phillips et al., 2014; Choi et al., 2017; Lei et al., 2019; Oogai and Nakata, 2021).

More recently, it has been shown that sRNAs can also modulate gene expression by interacting directly with key regulatory proteins in the cell. In most Gram-negative bacteria, sRNA species act in concert with the bacterial protein Hfq (Massé et al., 2003; Gottesman, 2004; Sauer, 2013; Park et al., 2021) that may function by stabilization of RNA structures required for mRNA binding. However, it should be pointed out that riboregulation differs significantly between Gram-positive and -negative bacteria. Although Hfq is present in nearly 50% of all bacteria, its presence is much rarer in Gram-positive bacteria, and its core functions are not well understood in these bacteria. Additionally, Hfq is not universally present in all Gram-negative bacteria. Therefore, it is important to know what other proteins, if any, are required for efficient sRNA–mRNA interactions in bacteria that lack Hfq. Notably, some bacteria regulate sRNA through a different mechanism that involves binding of sRNAs to a translational inhibitor RNA-binding protein. The global regulators belonging to the Csr/RsmA family of RNA-binding proteins regulate multiple virulence determinants as well as other biological processes (Romeo et al., 2013; Kusmierek and Dersch, 2018). These proteins bind to 5′ untranslated regions of mRNAs and inhibit association of translational machinery (Duss et al., 2014; Vakulskas et al., 2015). Interestingly, sRNAs can antagonize the binding of Csr to mRNA by associating with its nucleotide-binding motif. This is due to the fact that both target mRNAs and antagonistic sRNAs carry the same consensus Csr-binding motif (Liu and Romeo, 1997; Liu et al., 1997; Wang et al., 2005; Wang et al., 2005). Recent genomic approaches are identifying large numbers of sRNAs, but their function is yet to be determined. Furthermore, Csr-/Rsm-like regulators have been identified in other bacteria, but their role in those organisms and molecular mechanisms are not known. Recently, a third RNA-binding protein, ProQ, which is involved in global regulation in Proteobacteria (such as *E. coli* and *Salmonella*) was reported (Smirnov et al., 2016; Smirnov et al., 2017). Whether they also act through sRNAs that antagonize them or inhibit translation remains to be determined.

As mentioned above, riboregulation has emerged as a potential key regulatory mechanism in *P. gingivalis*. However, although *P. gingivalis* is a Gram-negative bacterium, it lacks Hfq, thus leaving the mechanism of regulation by the sRNAs unknown. While searching for possible sRNA chaperones, we identified a small protein with the characteristics of an RNA-binding protein in *P. gingivalis* that we have designated RbpPg1 (RNA-binding protein *Porphyromonas gingivalis* 1). This finding is consistent with two reports showing that *Bacteroides*, in general, lack the major RNA-binding proteins, such as Hfq, ProQ, and CsrA, that are found in other bacteria (Adams et al., 2021; Prezza et al., 2022; Rüttiger et al., 2025). Instead, they code for proteins with K-homology (KH) motifs, RNA-recognition motifs (RRM), and cold shock domains (CSD). *Bacteroides thetaiotaomicron* has been shown to encode for many such RNA-binding proteins, which it uses to regulate polysaccharide metabolism (Adams et al., 2021; Rüttiger et al., 2025). However, *P. gingivalis*, a member of *Porphyromonadaceae* family, differs from *B. thetaiotaomicron* as it is an asaccharolytic organism that relies on peptides provided by the highly proteolytic protease repertoire found in this bacterium (Curtis et al., 1999a; Guo et al., 2010; Dubin et al., 2013). Here, our work further extends those findings and demonstrates that the RRM-1-like protein RbpPg1 binds RNA and is involved in maintaining metal homeostasis, Arg-X and Lys-X gingipain activity, response to hemin levels, and required for survival in the presence of host cells.

## Materials and methods

### Bacterial culture preparation

*Porphyromonas gingivalis* W83 was used as the parent strain for the study. The strain was maintained on blood agar plates (TSA with sheep blood, BBL) in an anaerobic chamber (80% N, 10% H, and 10% CO_2_). Broth cultures were prepared by inoculations of BHI broth supplemented with hemin (5.0 µg/ml) and vitamin K3 (1 µg/ml). Overnight cultures served as a starter for 1:10 dilutions used for cultures to be processed for further studies (growth studies, transcriptomics, and proteomics). Tetracycline or clindamycin (both 0.5 µg/ml) was used to select and maintain recombinant strains.

For metal sensitivity studies, the bacterial strains were inoculated to an OD_660_ of 0.1 and grown for 48 h in the presence or absence of metal salts. Bacterial growth was determined by measuring optical density at OD_660nm_.

*Escherichia coli (E. coli)* was grown aerobically at 37**°**C in Luria–Bertani (LB) broth (cat. no. 12780029, Invitrogen) or on LB agar. Kanamycin or carbenicillin (50 μg/ml) was added to select for recombinant strains.

#### Recombinant protein expression and RNA-binding studies

##### i) Recombinant RBP, HUβ, and HUα with His-Tag

Primers to amplify coding regions of the PG0627 encoding RbpPg1 and two genes coding for size-matching nucleic acid-binding proteins Huα (PG0121) and Huβ (PG1258) were designed (see **Supplementary Table 2**) that included restriction enzyme sites *Nco*I and *Xho*I allowing for cloning into pET30a expression vector (Novagen). The PG0121 and PG1258 were chosen as those that code for proteins predicted to bind single-stranded nucleic acids. Furthermore, the size of those proteins is similar to the RbpPg1, and thus, those were suitable controls for studies of the ability/specificity of proteins to bind RNA. PCR was performed using platinum PCR SuperMix (Invitrogen) with 35 cycles consisting of 94**°**C for 30 s, 52**°**C for 30 s, and 72**°**C for 3 min. Following product verification by gel electrophoresis, the PCR fragments were digested and cloned into the pET30a at the *Nco*I and *Xho*I sites. The recombinant plasmids were transformed into BL21 (DE3) competent *E. coli* cells for protein expression.

###### His-Tag purification of RbpPg1, HU-beta, and HU-alpha

1L BL21 cell cultures in LB media were induced with IPTG at an OD_660_ of 0.6 and incubated overnight at 37°C. Cultures were centrifuged into pellets and suspended in binding buffer [50 mM Na_2_HPO_4_, 300 mM NaCl, and 10 mM imidazole (IMD), pH 8.0]. Cells were lysed using Cell-Lytic B reagent (Sigma-Aldrich), the mixture was centrifuged, and supernatant was added to the Ni-NTA agarose (cat. No. 30210, Qiagen) for His-tagged purification by gravity-flow chromatography. The Ni column was washed with 100 ml of wash buffer (50 mM Na_2_HPO_4_, 300 mM NaCl, and 20 mM IMD), and His-tagged proteins were eluted with elution buffer (50 mM Na_2_HPO_4_, 300 mM NaCl, and 250 mM IMD). The eluted fractions were collected and run on NuPAGE**^®^** 12% Bis-Tris precast gel (Life technologies) to asses purity and concentration of the protein.

###### His-Tag repurification on Dynabeads

RbpPg1, HU-alpha, and HU-beta purified using nickel column were dialyzed in binding buffer overnight at 4°C, and the proteins were applied to the Dynabeads^®^ His-Tag Isolation and Pulldown (Novex), incubated for 1 h at 4°C, and supernatant was decanted. The beads were then washed four times with nuclease-free binding buffer, and proteins were eluted using nuclease-free elution buffer. The proteins were then dialyzed in nuclease-free binding buffer overnight at 4°C.

##### ii) Recombinant RBP, HUβ, and HUα with Halo-Tag

The pFC20K HaloTag plasmids (Promega) were used to clone PG0627, PG0121, and PG1258 at the *SgfI* and *PmeI* sites, thus generating pFC20K-RBP, pFC20K-HU-beta, and pFC20K-HU-alpha. The recombinant proteins were fused to HaloTag that binds covalently to HaloTag resin allowing for purification of recombinant protein. TEV protease cleavage site was engineered between the recombinant protein and the tag to release the recombinant protein from the resin during purification and obtain tagless recombinant protein. The plasmids were transformed into BL21 cells and plated on LB agar plates (containing kanamycin) to select for recombinant colonies.

###### HaloTag protein isolation

Cultures of BL21 (80 ml), carrying the respective recombinant plasmids, were induced with IPTG at an OD_660_ of 0.6 and incubated overnight at 37°C. The bacterial cells were then pelleted by centrifugation, washed with Purification Buffer (150 mM NaCl, 50 mM HEPES, and 1 mM TCEP, pH 7.4), and lysed with CelLytic B reagent (Sigma-Aldrich). The cell lysates were centrifuged at 12,000 rpm for 15 min, and supernatant was applied to HaLoLink resin (Promega). Following 45 min of incubation at room temperature, the resin-tagged protein was washed three times with 20 ml of RNase-free pulldown buffer (3.25 mM sodium phosphate and 70 mM NaCl, pH7.4), and the recombinant protein was released by incubation with 100 μl of TEV protease mix overnight at 4**°**C.

### Electrophoretic mobility shift assay

The Light Shift^®^ Chemiluminescent RNA EMSA Kit (Thermo-Fisher) was used to perform gel shift assays. The components of the binding reactions were assembled on ice according to protocol in the order listed in **Supplementary Tables 3**-**5**. Mixtures were incubated on ice for 30 min, and the products were resolved on Novex TBE 10% gel run at 100 V at 4°C. Electrophoretic transfer of the binding reaction to a nylon membrane was done under cooled 0.5× TBE buffer at 300 mA for 50 min. The nucleic acids were then crosslinked to the membrane, and biotin-labeled RNA was detected by chemiluminescence.

#### Protein–RNA pulldown

##### (i) His-tagged proteins

RNA was isolated from *P. gingivalis* W83 using the Qiagen RNeasy^®^ kit and treated with DNA-free™ kit (Ambion). The RNA was electrophoresed on an agarose gel to test for degradation. Of the *P. gingivalis* W83 RNA, 10 μg was then added to 25 μg of purified recombinant proteins containing His-tag: RbpPg1, HUα, and HUβ. The RNA–protein mixture was incubated for 1 h at 4°C on an inverter. The mixture was then applied to 50 μl of cobalt Dynabeads (Invitrogen). The pulldown was performed according to the Dynabeads protocol (Invitrogen) using nuclease-free buffers. The tube was placed for 2 min on a magnetic stand (to separate the beads from the supernatant). Following the removal of the supernatant, the beads were washed four times with binding buffer, and then His-elution buffer was added to remove the protein–RNA complexes. The elution was treated with protease (protease from *Streptomyces griseus*, Pronase E, Sigma, P8811), and following cleanup, released RNA was used for RNA library generation and sequencing.

##### (ii) Halo-tagged proteins

Halo-tagged proteins were pulled down using Halo-Link Resin (Promega), and 60 μg of *P. gingivalis* W83 RNA was added to each of the resin-tagged recombinant proteins. Following incubation at 4**°**C for 1 h, the resin was washed three times with 20 ml of RNase-free pulldown buffer (3.25 mM sodium phosphate and 70 mM NaCl, pH 7.4) and centrifuged at 2,000 rpm for 2 min. To release the protein–RNA complexes, 100 μl of TEV protease mix was added to each sample and incubated overnight at 4**°**C. To release RNA, the complexes were then treated with protease from *Streptomyces griseus* (Pronase E, Sigma, P8811). Briefly, 4.5 μl of 20 mg/ml of protease stock solution, 4.5 μl of 10% SDS, and 1.8 μl of 0.5 M EDTA were added to the protein–RNA elution and incubated at 50°C for 1 h. The RNA was cleaned with the RNeasy**^®^** kit (Qiagen), and the SMARTer**^®^** Stranded RNA-Seq Kit (Clontech^®^ Laboratories) was used to generate RNA-Seq library. The library was then sent to the VCU Genomics Core to be validated using the bioanalyzer and sequenced. High-throughput sequence reads were analyzed using the CLC Genomics Workbench software (Qiagen). Fold change was used to compare the reads from RBP with HU proteins.

###### Generation of recombinant *P. gingivalis* strains

The RbpPg1-deficient mutant was generated by replacing the gene encoding the RNA-binding protein (PG0627) with a gene encoding the clindamycin/erythromycin resistance in anaerobic bacteria, *ermF*. Briefly, a DNA fragment carrying the *ermF* gene was flanked by sequences adjacent to the PG0627 (150 bp on each side). The DNA fragment was PCR amplified and used to electroporate *P. gingivalis* W83 as described elsewhere (Fletcher et al., 1995). Mutant colonies were selected on BHI agar plates supplemented with 0.5 µg/ml of clindamycin. The colonies were characterized for the gene deletion/replacement by PCR/gel electrophoresis and sequencing. The mutant strain was designated as V3139. The mutation was restored using the pG108-*rbppg1* vector (Jones et al., 2020). Thus, the PG0627 gene and upstream regions were inserted into the pG108 vector, and the recombinant vector was electroporated into V3139. The mixture was cultivated overnight in BHI under anaerobic condition and plated on blood agar plates supplemented with tetracycline. Colonies appearing on the plates were verified by plating on blood agar supplemented with clindamycin and tetracycline as well as plasmid isolation and verification. The complemented strain was designated as V3236.

###### Determination of metal content in bacterial cells

Six milliliters of overnight cultures grown in BHI were pelleted and washed twice with Chelex-treated buffer (50 mM HEPES, 50 mM NaCl) to eliminate any extracellular metal content. The pellets were suspended in 3 ml of the Chelex-treated buffer supplemented with 8 M urea, sonicated (3 min at 20% duty cycle on ice), and centrifuged at 8,500 rpm for 30 min. The supernatant was removed for analysis via either inductively coupled plasma mass spectrometry (ICP-MS) or colorimetric ferrozine assay.

For ICP-MS, using argon gas, elements in the liquid samples were converted to ions, separated, and detected by a mass spectrometer (Wolf, March 2005). Results were given as relative abundance of individual metal ions (Mg, Fe, Co, Ni, Cu, Zn) in parts per trillion and compared to standards.

For the colorimetric ferrozine assay, 500 μl of the sample was incubated with 0.01 M ferrozine and 5 M ammonium acetate (buffer) for 5 h anaerobically. The intensity of purple color was determined by optical density readings at 562 nm. Relative abundance was quantified in parts per million from a standard curve.

### Protease assays

#### Arg-X activity

Five microliters of bacterial sample was aliquoted out into a VIS cuvette. Five hundred microliters of 1 M Tris-HCl pH 8.0 with 4 mM TCEP was added into each cuvette. A blank control (without bacterial supernatant) was also created. Baseline absorbance values were recorded to ensure all samples had an OD_405_ of 0.050. Fifty microliters of DL-BAPNA at a concentration of 4.3 mg/ml of DMSO was added to each cuvette including the blank. After 5 min, absorbance readings at 405 nm were taken, and the measurements were repeated every 15 min up to 45 min of the reaction assay.

#### Lys-X activity

For the Lys-X activity, the samples were prepared as above (for the Arg-x activity) except that 25 μl of D-Phe-Pro-Lys-pNA at a concentration of 4.9 mg/ml of DMSO was added to each cuvette to assess the gingipain protease activity. Protease activity was monitored by absorbance readings at 405 nm every 30 min up to 120 min of the reaction assay.

### Hemin depletion growth studies

An overnight culture in BHI media supplemented with vitamin K was diluted 1:10 in BHI media and grown until an OD_660_ of 1.0. A 1:10 dilution in mycoplasma media supplemented with vitamin K but without hemin was made and grown overnight. OD_660_ readings were taken prior to the next passage in the mycoplasma media without hemin. Passaging of bacterial samples continued every other day by making 1:10 dilutions using the mycoplasma media without hemin until OD_660_ was less than 0.1.

### Nitrosative stress assessment

Bacterial cultures (10 ml) were prepared using mycoplasma media, with and without 5 μg/ml of hemin, and passaged twice before being grown to an optical density at 660 nm of 0.05 anaerobically. Various concentrations of nitrosative stress-generating species were then added to the cultures as follows: nitrate (NaNO_3_; 40 and 20 mM) or nitrite (NaNO_2_; 8, 4, and 1 mM). Bacterial growth was monitored by measuring the OD_660_ using culture aliquots that were removed at various time points (0, 4, 8, and 16 h) and. Three sets of biological replicates (each done in technical triplicates) were conducted on different days to ensure significance of the results.

### Oxidative stress assessment

BHI agar plates were prepared (20 g/L of agar, 37 g/L of brain–heart infusion, 5 μg/ml of hemin, 5 mg/ml of yeast extract, 0.5 μg/ml of vitamin K, 1 mg/ml of L-cysteine) and allowed to equilibrate in an anaerobic chamber. Overnight cultures of *P. gingivalis* W83 and V3139 were diluted and allowed to grow to a final OD_660_ of 0.7. Two hundred microliters of 7 x 10 (Miyakawa et al., 2004) cells/ml of cell suspension was plated on the BHI agar plates. After 30 min, sterile filter disks were placed in the center of each plate, and 90 μl of either 1% or 5% of H_2_O_2_ solution (used as an oxidizing agent) was added to each filter disk. Plates were incubated in an anaerobic chamber at 37°C for 5–7 days, and the diameter of growth inhibition zones created around the discs was measured.

### RNAseq analysis

*P. gingivalis* strains W83 and V3139 were started from blood agar plates and incubated overnight in BHI. The cultures were diluted in BHI to OD_600_ = 0.1 and grown to mid-logarithmic phase. Dipyridyl was added to generate iron-deplete conditions. RNA from pelleted cells was isolated using the RNeasy mini-kit (Qiagen), and any residual DNA was removed using the DNA-free DNase kit (Ambion). RNA-seq library was generated using the Ovation Complete Prokaryotic RNA-Seq DR multiplex kit (Nugen), and quality of the libraries (fragment sizes from sonication during library generation) were confirmed using a bioanalyzer (Agilent Technologies). The libraries were submitted to the VCU Genomics Core and sequenced (pair-end sequencing 2 × 75 bp) on the Illumina MiSeq. The samples were deconvoluted and analyzed using the CLC Genomic Workbench 12.0.3. Sequences were imported into the software as paired-end reads, aligned to the *P. gingivalis* W83 genome NC_002950 using the RNA-Seq analysis tool, and then, statistical analysis for differential expression in two groups was performed. Four biological replicates prepared on separate days were used for the analysis.

### Bacterial culture preparation for proteomics analysis

*Porphyromonas gingivalis* W83 and V3139 (Δpg0627) were maintained on blood agar plates at 37°C in an anaerobic chamber. V3236 (Δpg0627 complementing strain) was grown in the same condition with 0.5 µg/ml of tetracycline on the blood agar plate. For liquid cultures, strains were inoculated into 3 ml of BHI and incubated overnight at 37°C anaerobically (BHI supplemented with 0.5 µg/ml of tetracycline was used to cultivate V3236). The cultures were then inoculated into 17 ml of BHI (without any antibiotic added) and grown anaerobically at 37°C for 24 h. The following day, the 20-ml cultures were inoculated into 80 ml of BHI without any antibiotic added and grown at 37°C in anaerobic conditions. Pellets were collected when the culture OD_600_ reached 0.7. The samples were prepared on four different days, thus generating four biological replicates. Four tubes of 10 OD_600_ of culture per sample were frozen in −20°C and sent to the Center for Proteomics Discovery at John Hopkins University for proteomics analysis.

### Proteomics analysis

#### Sample preparation

Bacterial cells were suspended in a lysis buffer composed of 8 M urea/50 mM tetraethyl ammonium bicarbonate. Released proteins were digested with enzymes: LysC (LysC-to-protein ratio of 1:100) and trypsin (trypsin-to-protein ratio of 1:100). The samples were then labeled with 10-plex Tandem Mass Tagging TMT. Finally, samples were fractionated (12 fractions) using the basic pH RPLC method.

#### Mass spectrometry analysis

Samples were analyzed on the Orbitrap Fusion Lumos (Thermo Scientific) with an on-line liquid chromatography: easy nLC 1200 (Thermo) with a 2-h running time per fraction. MS1 resolution was 120,000, and MS2 resolution was 30,000. HCD fragmentation method was used, and collision energy for MS2 was 32.

#### Database search

The Proteome Discoverer 2.1 software was used as our database search tool. The *Porphyromonas gingivalis* (strain W83) UniProt (released on April 2017) was used with two missed cleavages allowed. Cleavage enzyme: trypsin, minimum AA length: 6, minimum peptides for protein: 1, fixed modification: carbamidomethyl on cysteine residue and TMT on Lys and peptide N-terminal, dynamic modifications: oxidation on M and acetyl on N-terminus. A 1% false discovery rate was applied for both peptide and protein levels.

#### Statistical analysis

The software Perseus was used for analysis (Cox and Mann, 2012). p-Value was calculated using Student’s t-test. q-Value was calculated using significance analysis of microarrays (SAM) (Tusher et al., 2001) and permutation-based false discovery rate (FDR). s0 value for SAM: 0.1 Reference for SAM. NOTE: q-value is a modified p-value that is more stringent accounting for multiple hypotheses. For q-value calculation, both p-value and fold change were considered. Proteins with q-value lower than 0.05 are considered to be statistically significant in our study.

### Host–pathogen interaction studies

#### Bacterial survival study

An immortalized oral epithelial cell line, HN4 cells (Miyazaki et al., 2006a), and primary human oral keratinocytes (HOKs) (ScienCell) were used for our study, and infection was carried out as described previously (Lewis et al., 2012; Sengupta et al., 2014; Wunsch and Lewis, 2015; Belvin et al., 2019). HN4 cells were cultured in DMEM + 10% FBS, and HOKs were cultured in human oral keratinocyte medium (ScienCell) in a 37°C, 5% CO_2_ incubator. Briefly, cells grown to confluence in flasks were plated in 12-well tissue culture plates at approximately 50,000 cells per well. The cell culture medium was rendered anaerobic by maintaining it in anaerobic chamber overnight. Prior to infection, the HOK-containing plate was placed in an anaerobic chamber, and media were replaced with the anaerobic cell culture medium. The cells were infected with wild-type *P. gingivalis*, RbpPg1-deficient strain V3139, or the complemented strain V3236 at a multiplicity of infection of 100:1. For total survival, the cells were incubated for 1 h under an atmosphere of 6% oxygen at 37°C, washed with PBS, lysed with 1% saponin to release intracellular bacteria, and serial dilutions of the lysate were plated on TSA blood agar plates. To account for intracellular bacterial survival only, gentamicin/metronidazole (300 μg/ml of gentamicin and 400 μg/ml of metronidazole) was added to the infected cells to kill the extracellular bacteria. Following 1 h of the antibiotic treatment, the cells were washed, lysed, and plated as above. Colonies that appeared after 5–8 days were counted to calculate the colony-forming units (CFU)/ml for each infection.

#### Attachment and microbial internalization—flow cytometry analysis

Total interaction and internalization of *P. gingivalis* strains into eukaryotic non-phagocytic cells using human umbilical vein endothelial cells (HUVEC) was determined through fluorescent tagging of bacteria with Fluorescein Isothiocyanate (FITC; Sigma, St. Louis, MO), quenching via trypan blue, and analysis via flow cytometry (BD FACS Canto II, Becton Dickinson, Franklin Lakes, New Jersey). Three milliliters of bacterial cultures (OD660 readings between 0.4 and 0.7) was pelleted, washed twice with PBS anaerobically, and suspended in 1 ml of PBS. Two microliters of FITC was added to samples followed by 1-h incubation at 4°C in the dark. After incubation, samples were washed twice more with PBS under anaerobic conditions and suspended in 2 ml of HUVEC media. Infection was set up with a multiplicity of infection (MOI) of 100 bacterial cells to 1 HUVEC (100:1) and allowed to incubate anaerobically for 30 min at 37°C. Fluorescence was measured using the BD FACS Canto II flow cytometer equipped with an argon ion laser having a single excitation wavelength of 488 nm, with computer-assisted data analysis. FITC fluorescence was captured (emission wavelength of 518 nm) and plotted against the number of cells for a calculation of total interaction.

Trypan blue (100 µg/ml) acted as an extracellular quencher, thereby measurements were only accounting for internalized bacteria. Samples were incubated with trypan blue for 2 min. At least 10,000 cells were captured and analyzed for each sample for each of the interaction and invasion readings. Healthy cell populations were gated and analyzed using the FCS Express software, and gates were set to eliminate any unstained material or debris. Median was used as a measurement of relative absorbance of FITC for analysis and plotted in a graph.

## Results

### Identification of RbpPg1 using bioinformatics analysis

**Figure 1:**
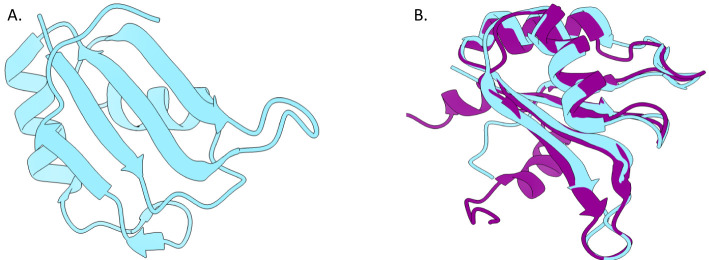
RbpPg1 has characteristics of an RNA-binding protein. **(A)** Crystal structure of RbpPg1 (encoded by PG0627 in the *P. gingivalis* W83 genome. PDB ID: 7JLY. Q7MWI3). **(B)** The structures of RbpPg1 (teal) and the N-terminal RRM domain of Cst-64 from *Homo sapiens* (PDB ID: 1P1T). The structures are overlayed with an RMSD of 1.067 Å across residues 16–78 of RBP.

The *P. gingivalis* W83 genome codes for a small 291-bp gene resulting in 97-aa protein (MW = 11,484) that contains a region (residues 3 to 82) known as an RNA-recognition motif or RRM (a.k.a. RNP or RBD domain). Based on those characteristics, we designated the protein as RNA-binding protein *P. gingivalis* 1 (RbpPg1). This protein has an RNA-binding fold similar to the *Homo sapiens* N-terminal RRM domain of cleavage stimulation factor 64-kDa subunit (Cst-64, PDB id:1P1T NMR structure, E-value 6.3 E −12) and consists of four antiparallel β-strands forming a β-sheet structure that is supported by two α-helices (**Figures 1A, B**) (Pérez Cañadillas and Varani, 2003). It is a negatively charged protein with a net charge of −1.76 and a pI of 5.40 but has a highly positively charged region across the surface of the β-sheet. The RMM motif facilitates protein–RNA binding that occurs across the face of the β-sheet via contacts between positively charged residues and the negatively charged backbone of RNA as well as base-stacking interactions with aromatic side chains. The RRM-1 motif is an N-terminal single ferredoxin-like motif followed by a disordered C-terminal region of different lengths (depending on the RRM-1 protein). Residues 4–82 of RbpPg1 are composed of two beta–alpha–beta folds that represent the ferredoxin-like motif that is found in variety of eukaryotic RNA-binding proteins and has recently been reported to be present in *Bacteroides thetaiotaomicron* as well as other prokaryotes (Adams et al., 2021; Mozumdar and Roy, 2024; Rüttiger et al., 2025).

An InterproScan of the PG0627 gene reveals that residues 3–82 represent a nucleotide-binding domain with an alpha–beta plate structure, which is consistent with the RRM motif found in RNA-biding domains of various ribonucleoproteins (**Supplementary Figure 1**) (Bochkarev et al., 1995; Kielkopf et al., 2004). Three separate databases indicated that the PG0627 protein sequence is indeed an RNA recognition motif domain as follows: 1) Pfam, residues 5–74; 2) SMART, residues 4–77; and 3) PROSITE, residues 3–81.

**Figure 2:**
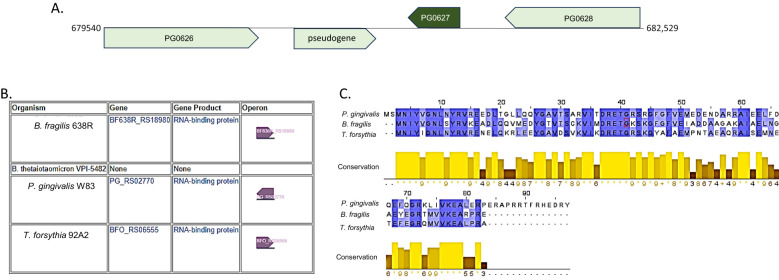
RbpPg1 is a unique RNA-binding protein. **(A)** Locus of PG0627 in the *P. gingivalis* W83 genome. The PG0627 gene comprises a single transcriptional unit, color coded in dark green. The gene coordinates are: 681,232–681,525, and it is bordered by a 462-bp pseudogene (PG0627 RS02770) downstream (coordinates: 680,600–681,061) and a gene PG0628 (RS02775) coding for a putative LPS export ABC transporter (coordinates: 681,774–682,529). **(B)** Orthologs of PG0627 in other Bacteroidota. **(C)** Protein sequence alignments of the PG0627 orthologs with conservation scores (1 indicates no conservation at site, an * represents a side that is completely conserved.

We also examined the genomic locus of PG0627 (PG_RS02770). As shown in **Figure 2A**, the gene is separated by 171 bp from a downstream pseudogene and 249 bp upstream from the PG0628 gene. It is transcribed as a single transcriptional unit. Of interest is the gene upstream of PG0627, designated PG0628, encoding an LPS export ABC transporter ATP-binding protein. A search for orthologs in other bacteria identified a similar protein in *B. fragilis* and *T. forsythia* but not in *B. thetaiotaomicron* (**Figure 2B**). Thus, the RBP identified in our studies differs from the one reported to regulate glycan utilization in *Bacteroides* and, thus, represents a novel class of this group of regulatory proteins. Also, the *P. gingivalis* RBP is longer than the orthologs in other bacteria with 97 aa vs. 81 aa, respectively (**Figure 2C**). BLAST search for similar protein in *Porphyromonas gingivalis* and other bacterial organisms has shown that the protein is conserved in *P. gingivalis* strains. It is also present in *Tannerella forsythia* and *Bacteroides fragilis* strains. However, no significant similarity was identified while searching the protein database of *Bacteroides thetaiotaomicron.* These data indicate that the protein is unique for the Bacteroidota phylum. As noted above, the protein also exists as a domain of multiple larger proteins in eukaryotic cells (Takagaki and Manley, 1997; Campagne et al., 2023; Schmeing and Hart, 2024). It is noteworthy that this protein is highly expressed in the available RNAseq and proteomics datasets.

### RbpPg1 binds RNA

**Figure 3:**
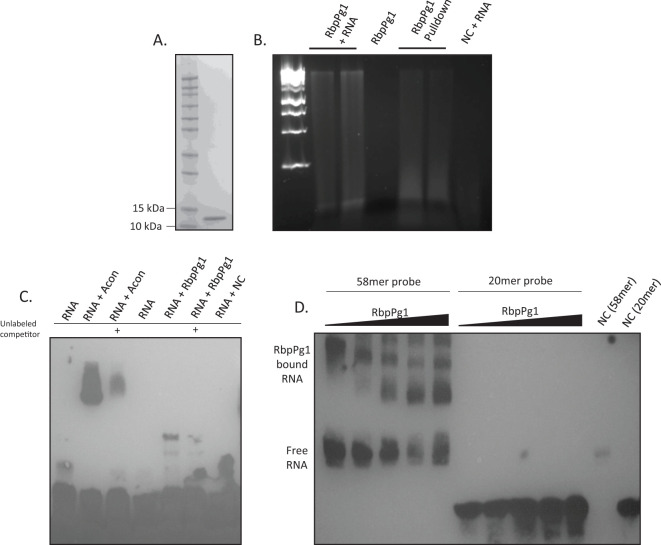
RbpPg1 binds RNA. **(A)** SDS-PAGE gel of purified RbpPg1. **(B)** RbpPg1 was incubated with whole RNA extract from *P. gingivalis*, or RNA eluted from His bound RbpPg1 was run on an agarose gel containing red dye detecting nucleic acid. Cytochrome C mixed with RNA was used as a negative control. **(C)** Electro-mobility shift assay (EMSA) of RNA fragment specific for IRE (iron-responsive protein). Cytosolic aconitase was used as positive control for RNA binding in lanes 2 and 3. RbpPg1 bound to IRE RNA is shown in lanes 5 and 6. An unlabeled probe was used as a competitive inhibitor of RNA binding. Cytochrome C was used as negative control for RNA binding in lane 8. **(D)** EMSA RNA probes synthesized for this study. A long 58-nucleotide probe containing the IRE stem loop structure, and a short 20-nucleotide probe lacking the IRE stem loop structure were incubated with Increasing amounts of RbpPg1 and run on a TBE page gel. Lane 11—long RNA probe incubated with cytochrome c, lane 12—short RNA probe incubated with cytochrome c.

We prepared a recombinant form of RbpPg1 using *E. coli* carrying pET30-*rbppg1* (**Figure 3A**). The RbpPg1 was incubated with total RNA isolated from *P. gingivalis*, and RbpPg1–RNA complexes were pulled out with nickel-coated magnetic beads. As shown in **Figure 3B**, we were able to pull RNA with the PgRBP1 protein but not with the negative control protein cytochrome c, known to lack RNA-binding ability.

We then confirmed the binding using an RNA EMSA assay. As bioinformatics analysis of RbpPg1 has shown similarity of RbpPg1 to RRM-like proteins, which included the IRE (iron-responsive element) proteins, we first performed RNA EMSA using the RNA probe containing the IRE recognition motif. As a control, a cytosolic liver extract containing cytosolic aconitase that binds RNA was used. Cytosolic aconitase is an IRE-binding protein, which may regulate translation of mitochondrial aconitase mRNA (Zheng et al., 1992). As shown in **Figure 3C**, the IRE probe shifted when incubated with the IRE protein or with RbpPg1, thus confirming that RbpPg1 binds RNA. Reduced IRE RNA shift was observed for both IRE and RbpPg1 proteins when unlabeled specific competitor RNA was included in our reaction, thus indicating that the binding is specific. We then performed additional studies using other RNA oligos synthesized for this study. We used 50-nucleotide-long RNA oligo (containing the IRE motif as well as other protein recognition motifs) as well as short 20-nucleotide RNA oligo devoid of the IRE motif (**Figure 3D**; **Supplementary Table 2**—RNA sequences). A dose-dependent RNA shift was observed with increasing amounts of PgRbp1 for the 50mer RNA oligo, but no protein binding was detected with the short 20mer RNA oligo. This indicates that RbpPg1 may bind to the stem loop structures similar to those found in the IRE RNA. Furthermore, no RNA shift was observed with cytochrome c, a protein that does not bind RNA. Overall, these data demonstrate that RbpPg1 binds RNA.

**Figure 4:**
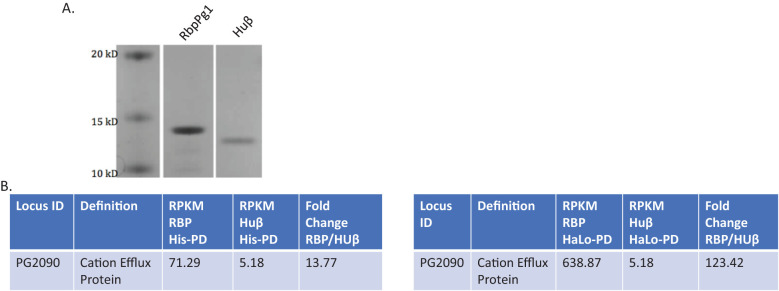
RbpPg1 RNA pulldown identifies zinc efflux transcript. **(A)** PAGE gel of RbpPg1 (lane 2) and Huβ (lane 3) eluted from HaLo-Resin resin after pulldown of whole *P. gingivalis* RNA. **(B)** The Cation Efflux protein encoded by PG2090 was the top hit from both His and HaLo RbpPg1 pulldown-RNAseq analysis.

To gain insight into the identity of RNA fragments that RbpPg1 is binding to, we performed RNA pullout using the following two forms of recombinant RbpPg1: His tagged and Halo tagged (**Figure 4A**). To test for enrichment of RNA samples, we utilized a *P. gingivalis* DNA-binding protein, Huβ, as a negative control. There were several RNA fragments that matched gene sequences that were enriched in the RbpPg1 pulldown samples compared to control HUβ pulldowns. As shown in **Supplementary Tables 6**, **7**, the most consistently drastically enriched gene was PG2090 coding for metal efflux protein (**Figure 4B**). There was 71.29-fold enrichment over the control HUβ protein when using His-tagged RbpPg1 and 638.87-fold when using HaLo-tagged one. More detailed analysis of the transporter designates it as a zinc transporter. The transporter is a 302-aa protein (909 bp) and has an integral membrane domain (19–219 aa, Pfam PF01545) and a ZT_dimer: dimerization domain of zinc (223–300 aa, Pfam PF16916). The proteins are designated as efflux pumps and increase the tolerance to divalent metal ions, such as cadmium, zinc, and cobalt. The C-terminal dimerization domain is found in zinc exporters that is located in cytoplasm and facilitates homodimer formation. The domain shares similarity to the metallochaperone Hah1 (UniProtKB:O00244). Among other enriched RNA sequences were the ones matching the genes of *hmuR*, magnesium transporter, ABC transporter (**Supplementary Tables 6**, **7**).

### RbpPg1 reduces zinc toxicity and alters metal content in *P. gingivalis*

**Figure 5:**
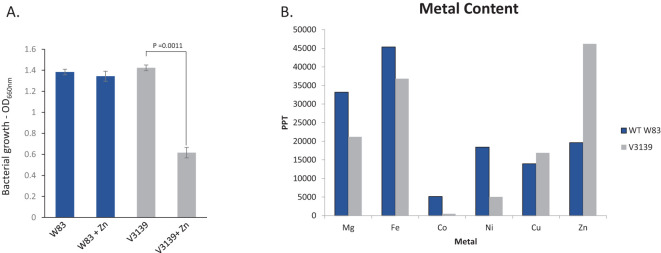
RbpPg1 contributes to sensitivity of *P. gingivalis* to zinc toxicity. **(A)** Sensitivity of *P. gingivalis* strains to Zn. Strains were grown in BHI broth with and without 0.5 mM ZnCl_2_ for 48 hr Data are derived from an experiment done three times on different days. **(B)** Intracellular metal content via ICP-MS of W83 and V3139. Values are displayed in parts per trillion.

Based on our findings that the RNA may bind zinc exporter and thus alter sensitivity of the bacteria to zinc, we generated an isogenic mutant deficient in the RNA-binding protein and assessed its sensitivity to minimal concentration of ZnCl_2_. As shown in **Figure 5A**, wild-type *P. gingivalis* W83 can withstand a high concentration of zinc as 0.5 mM was not inhibitory. However, approximately 55% reduction in growth was observed in the mutant V3129 devoid of RbpPg1, thus indicating that the protein affects the microbial tolerance to zinc. We next examined the metal content in the parental *P. gingivalis* W83 and the mutant V3139 strains. As shown in **Figure 5B**, there was over a 2.5-fold increase in intracellular Zn in the V3139 strain when compared to the wild type. This corresponds with a decrease in content of the following metals seen in V3139: a 1.57-fold decrease in magnesium, a 1.23-fold decrease in iron, a 10.62-fold decrease in cobalt, and a 3.66-fold decrease in nickel. Concurrently, there was a 1.21-fold increase in copper.

We further examined the iron content of the cells using ferrozine assay. As shown in **Supplementary Figure 2**, V3139 in iron excess conditions had a 1.22-fold decrease in intracellular iron compared to W83. In iron-depleted conditions (through the addition of 2,2-dipyridyl or DP), V3139 saw a similar 1.18-fold decrease compared to the W83 strain.

### RbpPg1 facilitates Arg-X and Lys-X protease activity

**Figure 6:**
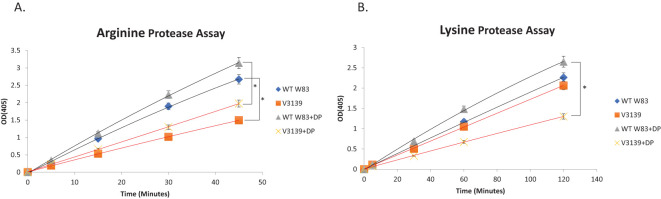
RbpPg1 is required for protease activity. Whole cell protease assays of Arg-X **(A)** and Lys-X **(B)** proteases using WT W83 and the RbpPg1-deficient strain V3139. Protease activity was monitored via the degradation of BApNA and D-Phe-Pro-Lyz-pNA at 405 nm. Three biological replicates were done, and paired Student t-test and p-values <0.05 were used to analyze the data. Significant difference is denoted by an asterisk "*".

The gingipain family of proteases are a major virulence factors in *P. gingivalis*; however, how their expression and activity are modulated is not fully understood (Curtis et al., 1999a). Thus, we examined the effect of RbpPg1 deletion on both Arg-X and Lys-X protease activity using cell culture in both iron-excess and -limited conditions. As shown in **Figure 6A**, in iron-excess conditions, V3139 displays a 1.79-fold decrease in arginine protease activity compared to W83. Similarly, in iron limited conditions, V3139 shows a 1.59-fold decrease in protease activity compared to W83. Overall, for both strains, arginine protease activity was greater in iron-limited conditions than in iron excess conditions, but the RbpPg1 enhances Arg-X protease activity under both conditions. Similar to that seen above, W83 showed a greater lysine protease activity in iron-limited than in iron-excess conditions (**Figure 6B**). However, V3139 had a significant decrease in lysine protease activity in iron-limited conditions compared to that in iron-excess conditions (**Figure 6B**). Additionally, in iron-excess conditions, V3139 showed a 1.10-fold decrease in activity compared to W83. However, in iron-limited conditions, V3139 had a 2.04-fold decrease in lysine protease activity compared to W83.

In conclusion, RbpPg1 enables both Arg-X and Lys-X protease activity. The differences are significant as assessed using the paired Student t-test with p-values below 0.05. Of note was also the observation that Arg-X protease activity is significantly higher than the Lys-X protease activity across all strains grown in either iron-excess or iron-limited conditions.

### RbpPg1 modulates *P. gingivalis* growth under hemin-deplete conditions

**Figure 7:**
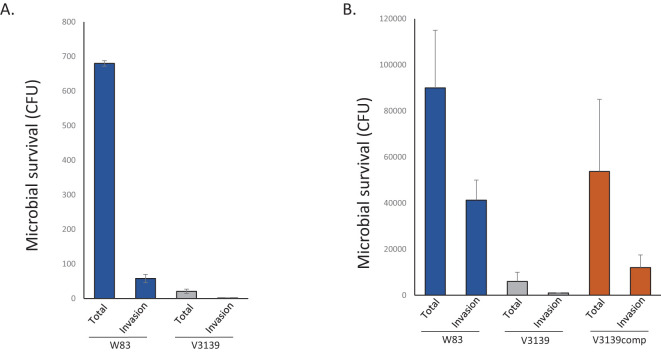
RbpPg1 is required for survival of *P. gingivalis* with host cells. **(A)** Survival of WT W83 *P. gingivalis* and V3139 RbpPg1-deficient strain with the immortalized HN4 cell line. Bacterial survival was quantified by counting colony-forming units following lysis of infected cells. MOI used was 100:1. **(B)** Human oral keratinocytes (HOKs) infected with WT W83, V3128-*rbpPg1* mutant, and complemented strain (V3236) at an MOI of 100:1.

**Figure 8:**
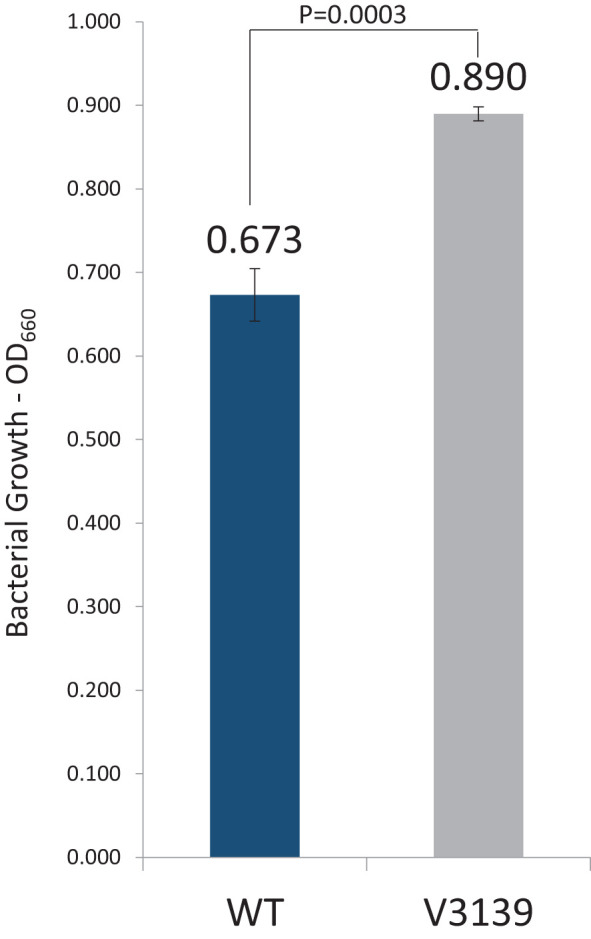
Loss of RbpPg1 enhances growth under hemin-limited conditions. Samples were passaged in Mycoplasma media without hemin. Data are from the third passage. The number displayed above the respective bars shows the OD_660_ value. Data are representative of three biological replicates. p-Value represents paired Student t-test.

The graph in **Figure 7** illustrates the growth of V3139 versus W83 strain in hemin-depleted conditions. A decrease in growth was expected in W83 as hemin, the iron-containing protoporphyrin, is an essential nutrient for growth of *P. gingivalis*. However, an unexpected occurrence was the high growth of V3139 under conditions of low hemin. At day 0, all samples were grown to an OD_660_ of 1.0 in BHI supplemented with vitamin K. The first 1:10 passage in mycoplasma without hemin was conducted, and an OD_660_ was taken 2 days later just prior to the second passage. This continued through day 8 when the optical density fell below 0.1 for both strains (not shown). Data from day 6, or the third passage, showed statistically different growth and is depicted in **Figure 8**. V3139 grew better in a hemin-depleted state compared to W83, as the OD_660_ reading measure at 0.890 for V3139 compared to that of W83 was at 0.673. This translates to a 33% decrease in growth of W83 and an 11% decrease in growth in V3139.

### RbpPg1 plays no role in growth of *P. gingivalis* with nitrosative stress

Effective nitrosative stress protection mechanisms are needed for *P. gingivalis* to thrive in the oral cavity. This study utilized various concentrations of nitrosative stress-generating compounds including NaNO_3_: 40 and 20 mM and NaNO_2_: 8, 4, and 1 mM. Samples were grown in mycoplasma media supplemented with hemin or without hemin, and growth was monitored with OD_660_ taken at 0, 4, 8, and 16 h. For both strains, there was no considerable difference in growth inhibition between 40 mM NaNO_3_ and 20 mM NaNO_3_ (**Supplementary Figures 3A, B**). Growth in NaNO_2_ yielded similar results, with no significant difference in growth between the W83 and V3139 strains. V3139 displays a small growth inhibition difference between 1 and 4 mM NaNO_2_, while the wild-type shows a slightly larger effect between the two in terms of growth inhibition, but this effect was not statistically significant (**Supplementary Figures 3C, D**). Notably, the increased growth of the RbpPg1 mutant strain seen in heme-limited conditions persists under nitrosative stress conditions.

### RbpPg1 has no effect on peroxide stress in *P. gingivalis*

This disk diffusion study utilized cultures in logarithmic phase of growth that were plated on BHI-agar plates. The cultures were then overlayed with disks saturated with two concentrations of hydrogen peroxide (1% and 5%), and the cultures were grown in an anaerobic chamber at 37°C for 5–7 days. Diameters of the zones of growth inhibition were then measured for each plate. As shown in **Supplementary Figure 3B**, there was no significant difference in the zone of inhibition between WT W83 and V3139 at 1% or 5% H_2_O_2_.

### Deletion of RbpPg1 reduces survival of *P. gingivalis* with host cells

The oral epithelial cell line HN4 (Miyazaki et al., 2006a; Miyazaki et al., 2006b) was first used to gain insight into the effect of RbpPg1 mutation on survival of *P. gingivalis* with host cells. HN4s were exposed to *P. gingivalis* strains for 30 min under anaerobic conditions (Wunsch and Lewis, 2015). To determine the survival of internalized bacteria, extracellular bacteria were killed by treatment of the infected cells with metronidazole. We observed a 10-fold reduction in survival of the RbpPg1-deficient strain in the presence of host cells (survival of both attached and internalized bacteria) when compared to the parental W83 bacteria (**Figure 7A**). These results indicate reduced ability of the RbpPg1-deficient strain to survive with host cells. To ensure that survival rather than attachment/internalization was affected, we also performed flow cytometry analysis that showed that both strains have the same potential to associate with eukaryotic cells (**Supplementary Figure 4**).

In addition, primary human oral keratinocytes (HOKs) were used to determine the effect of *rbppg1* deletion on survival with host cells. For these assays, the V3139 strain was complemented by providing a copy of the *rbppg1* gene on a plasmid. As shown in **Figure 7B**, drastic reduction in survival of the bacteria with host cells is observed. Both total survival and survival of internalized bacteria are observed in the mutant strain when compared to the parental W83 strain. The ability to survive with the host cells was restored when a complete copy of the gene was provided on a plasmid (strain V3236). As survival of *P. gingivalis* with host cells is required for virulence of the bacteria, we conclude that the virulence potential of RbpPg1-deficient strain is significantly reduced.

### RNAseq analysis

Analysis of the differential regulation of genes resulting from inactivation of RbpPg1 resulted in several deregulated candidates. The results are shown in **Supplementary Tables S8**, **S9**. The main reduction in transcript reads in the mutant strain was for PG0627. This would be expected as it was the deleted gene in the V2329 strain, and it codes for the RbpPg1 protein. There was moderate regulation of other candidates, but none was significant indicating that RbpPg1 is not involved in deregulation at a transcriptional level.

### Proteomics analysis

**Figure 9:**
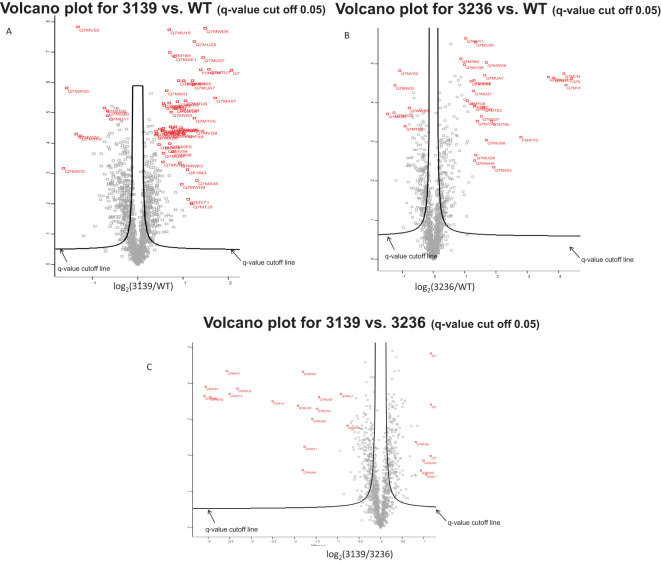
Effect of RbpPg1 on proteome alteration in *P. gingivalis* W83. Differential expression of proteins between V3129 and WT **(A)**, V3236 and WT **(B)**, and V3139 and V3236 **(C)**. In red font are the highlighted accession numbers of proteins with high confidence of differential expression. The proteins outside the q-value cutoff line can be considered to be significant with a 5% of possibility being false positive.

As RNA-binding proteins are expected to play a role in RNA metabolism and modulation of protein expression to a higher degree than regulating transcript abundance, we performed high-throughput quantitative proteomics on whole cell lysates. Protein content was examined in the parental W83 strain, the *P. gingivalis* V3139 (RbpPg1—deficient strain) and the *P. gingivalis* V3236 (RbpPg1—overexpression strain). In total, 1,393 total proteins were identified (**Supplementary Table 10**). This is excellent coverage of the *P. gingivalis* proteome that is composed of approximately 1,916 proteins. A total of 592 proteins were found to be differentially regulated when comparing the protein abundance in W83 and the V3139 strains (graphical representation of the regulated proteins is shown in **Figure 9**). The most drastically upregulated proteins in the mutant strain, V3139, are listed in **Table 1** (all differentially regulated proteins are listed in **Supplementary Table 11**) and include oxidative/nitrosative stress defense proteins. Among the upregulated ones are SigH (PG1827), ferritin (Ftn), thiol peroxidase (Tpx), thioredoxins (Trx, TrxB), superoxide dismutase (Sod), thiol protease (Tpr), glycerate dehydrogenase (HprA), alkaline phosphatase (PG0890), formate tetrahydrofolate ligase (Fhs), acetyltransferase (GNAT family, PG1842), and FeoB2. Reduction in transcription reads was observed for the RNA-binding protein (Rbp, PG0627), transcriptional regulator TetR (PG1240), RagA and RagB, alkylhydroperoxide reductase (PG0618-19), multiple helicases (PG1038, PG2047, PG1303), ribonuclease (Rnc), acyltransferase (PG1355), glycosyltransferase (PG0106), PorT, anaerobic ribonucleotide triphosphate reductase (PG1260), and single stranded DNA specific exonuclease (RecJ). Thus, stress response mechanisms were upregulated, while metabolic enzymes and protein transporters were downregulated.

**Table 1:** List of the most regulated proteins Mutant vs WT.

p value	Fold change (3139/WT)	Accession	Description
0.00067318	0.323635471	Q7MWI3	RNA-binding proteinPG_0627
1.5254E-06	0.339449698	Q7MXG5	Inner membrane proteinPG_0224
4.9838E-05	0.391878239	Q7MWZ4	Uncharacterized protein PG_0434
1.4847E-08	0.400980099	Q7MVS2	McrBC restriction endonuclease system, PG_0971
5.8643E-05	0.417820199	Q7MTF0	CRISPR-associated helicase Cas3 cas3
0.11249683	0.440240349	Q7MX17	Uncharacterized protein PG_0409
0.00022457	0.50971182	Q7MTH1	Uncharacterized protein PG_1988
0.05559325	0.52213587	Q7MXA4	Electron transport complex subunit D PG_0305
0.00540132	0.556686416	Q7MUX2	Acyltransferase, putative PG_1355
4.9141E-05	2.017508285	F5HFX8	TonB-dependent receptor, putative PG_2008
8.7342E-07	2.019118812	Q7MVE3	ATP:cob(I)alamin adenosyltransferase, putative PG_1124
0.02587896	2.032294729	Q7MU20	Uncharacterized protein PG_1752
4.0154E-06	2.075536238	Q7MTU6	Acetyltransferase, GNAT family PG_1842
0.00073974	2.143351585	Q51843	Pyridoxine 5'-phosphate synthase pdxJ
0.00699524	2.154916526	Q7MV71	Uncharacterized protein PG_1219
0.01011981	2.294362823	Q7MTJ5	50S ribosomal protein L33 rpmG
8.816E-07	2.30451165	P19665	Superoxide dismutase [Mn/Fe] sodB
1.207E-06	2.329659806	Q7MUA7	Uncharacterized protein PG_1625
4.4302E-08	2.368572013	Q7MUZ8	Formate--tetrahydrofolate ligase fhs
1.4844E-05	2.370698545	Q7MTV5	RNA polymerase sigma-70 factor, ECF subfamilyPG_1827
3.6548E-05	2.401412694	Q7MVQ9	Uncharacterized proteinPG_0987
0.00170786	2.463126971	Q7MX08	Uncharacterized protein PG_0419
3.8219E-07	2.614240637	F5HE54	Ferritin ftn
1.5172E-07	2.720508497	Q7MU37	Probable thiol peroxidase tpx
1.6447E-08	2.80073437	Q7MWD6	Uncharacterized protein PG_0686
3.7587E-07	2.953196005	Q7MTU7	Uncharacterized protein PG_1841
3.2653E-06	3.252988046	Q7MX07	Uncharacterized protein PG_0421
4.0134E-07	4.168044923	Q7MT60	Transcriptional regulator, AraC family PG_2125

Overexpression of RbpPg1 by 3.8-fold using V3236 (V2329 strain carrying PG108—*rbppg1*) resulted in the regulation of 228 proteins in V3236 when compared to that of the wild-type strain (the most regulated proteins are shown in **Table 2**, all statistically significant regulated proteins are shown in **Supplementary Table 12**). Upregulated proteins included proteins involved in hemin uptake (Hmu system), transcriptional regulators, TetR (PG1181) and AraC (PG2125), RNA sigma factor SigH (PG1827), formate tetrahydrofolate ligase (Fhs), ferritin (Ftn), thiol peroxidase (Tpx), thioredoxins (Trx, TrxB, PG1638), superoxide dismutase (Sod), thiol protease (Tpr), glycerate dehydrogenase (HprA), alkaline phosphatase (PG0890), acetyltransferase (GNAT family, PG1842), TonB-like protein (PG0631), and ferredoxin-like protein (PG1421). Among the downregulated were the following proteins: glycosyl transferase (PG0106, PG0110), pyruvate flavodoxin oxidoreductase (PFOR, PG0548), glycosyltransferase (PG1140), alkylhydroperoxide reductase (PG0619), multiple uncharacterized proteins, FeoB2, and RagA.

**Table 2:** **List of the most regulated proteins complemented vs WT**.

p value	Fold change (3236/WT)	Accession	Description	
0.00019	0.362503	Q7MXG5	Inner membrane protein PG_0224	
0.00018	0.418382	Q7MWZ4	Uncharacterized proteinPG_0434	
3.64E-05	0.423739	Q7MW02	Uncharacterized protein PG_0866	
0.009567	0.435012	Q7MWP8	Pyruvate-flavodoxin oxidoreductase PG_0548	
1.49E-05	0.473622	Q7MVS2	McrBC restriction endonuclease system, PG_0971	
0.000399	0.530723	Q7MTK6	Uncharacterized protein PG_1945	
0.036404	0.551925	Q7MT71	Phosphomethylpyrimidine synthase thiC	
0.004107	0.558137	Q7MTI2	Uncharacterized protein PG_1977	
0.002578	0.565889	Q7MUT1	Rhomboid family protein PG_1403	
0.010617	0.569905	Q7MTF0	CRISPR-associated helicase Cas3 cas3	
0.003648	0.571614	Q7MXR2	Glycosyl transferase, group 4 family protein PG_0106	
0.004094	0.579822	Q7MVX3	Uncharacterized protein PG_0901	
0.004084	0.582839	Q7MWQ8	Aminoacyl-histidine dipeptidase pepD-2	
0.000134	0.583369	Q7MWB5	Transporter PG_0715	
0.000358	0.588017	Q7MUQ6	Uncharacterized protein PG_1439	
8.18E-05	2.00557	Q7MXE2	Uncharacterized protein PG_0257	
2.26E-06	2.034954	Q7MVY1	Alkaline phosphatase, putative PG_0890	
0.001066	2.090617	Q7MWP5	DNA-binding protein, histone-like family PG_0555	
0.080933	2.096875	Q7MUD2	DnaK suppressor protein, putative PG_1597	
8.86E-05	2.114444	Q7MTU6	Acetyltransferase, GNAT PG_1842	
0.000169	2.14508	F5HE54	Ferritin ftn	
0.000971	2.154187	Q7MWH8	ThiJ/PfpI family protein PG_0634	
0.010842	2.181998	Q7MUG6	Conserved domain protein PG_1555	
2.78E-05	2.31199	Q7MXW5	Thioredoxin trx	
0.000107	2.344765	P19665	Superoxide dismutase [Mn/Fe] sodB	
0.007619	2.373926	Q51843	Pyridoxine 5'-phosphate synthase pdxJ	
4.94E-05	2.446825	Q7MU37	Probable thiol peroxidase tpx	
2.68E-05	2.482305	F5HFX8	TonB-dependent receptor, putative PG_2008	
0.002992	2.501131	Q7MWH9	Uncharacterized protein PG_0633	
0.000121	2.506504	Q7MTU7	Uncharacterized protein PG_1841	
2.81E-06	2.525473	Q7MUZ8	Formate--tetrahydrofolate ligase fhs	
0.002155	2.561537	Q7MUG9	TonB-dependent receptor HmuR hmuR	
0.00029	2.648049	Q7MTV5	RNA polymerase sigma-70 factor, ECF subfamily PG_1827
0.000225	2.903575	Q7MX07	Uncharacterized protein PG_0421	
0.000133	3.036811	Q7MTS3	Methlytransferase, UbiE/COQ5 familyPG_1870	
1.97E-05	3.123188	Q7MUA7	Uncharacterized protein PG_1625	
9.45E-06	3.225689	Q7MWD6	Uncharacterized protein PG_0686	
0.000906	3.234076	Q7MUG8	CobN/magnesium chelatase family protein PG_1553	
0.000299	3.623585	Q7MT60	Transcriptional regulator, AraC family PG_2125	
0.004474	3.820848	Q7MWI3	RNA-BP PG_0627	
0.000756	6.966375	F5HFY5	HmuY protein hmuY	
2.19E-05	12.90093	Q7MWU2	Uncharacterized protein PG_0495	
2.29E-05	14.47347	Q7MTT3	Flavodoxin PG_1858	
1.79E-05	18.1304	Q7MVN2	Lipoprotein, PG_1019	
3.47E-05	18.8991	Q7MV97	Transcriptional regulator, tetR family PG_1181	
2.44E-05	21.28252	Q7MV99	Uncharacterized protein PG_1179	

Finally, comparison of *P. gingivalis* V3139 (RbpPg1—deficient strain) to V3236 (RbpPg1—overexpression strain) showed differential level with statistical significance for 276 proteins. A total of 130 proteins were upregulated in the absence of RbpBPg1, while 146 were downregulated (the most regulated proteins are shown in **Table 3**; all statistically significant regulated proteins are shown in **Supplementary Table 13**).

**Table 3:** List of the most regulated proteins Complemented vs Mutant.

p value	Fold change (3139/3236)	Accession	Description
0.000223	0.058633	Q7MV99	Uncharacterized proteinPG_1179
0.000123	0.059837	Q7MV97	Transcriptional regulator, tetR family PG_1181
0.000241	0.06456	Q7MVN2	Lipoprotein, putative PG_1019
4.53E-05	0.084703	Q7MWI3	RNA-binding protein PG_0627
0.000191	0.087958	Q7MTT3	Flavodoxin PG_1858
0.00014	0.100017	Q7MWU2	Uncharacterized protein PG_0495
0.000308	0.176127	F5HFY5	HmuY protein hmuY
0.000411	0.264331	Q7MUG8	CobN/magnesium chelatase family protein PG_1553
0.025269	0.286483	Q7MUK9	Uncharacterized protein PG_1499
4.73E-05	0.287015	Q7MTS3	Methlytransferase, UbiE/COQ5 family PG_1870
0.00563	0.295235	Q7MX17	Uncharacterized protein PG_0409
0.00096	0.333212	Q7MUG6	Conserved domain protein PG_1555
0.000502	0.360122	Q7MUG5	Uncharacterized protein PG_1556
0.00024	0.370732	Q7MUG9	TonB-dependent receptor HmuR hmuR
0.007248	0.519464	Q7MTV9	Uncharacterized protein PG_1823
0.000192	0.529932	Q7MUL1	DNA-binding protein, histone-like family PG_1497
0.117756	0.555161	Q7MXA4	Electron transport complex subunit D PG_0305
0.001463	0.58836	Q7MTH6	CRISPR-associated protein, TM1791 family PG_1983
0.085202	1.510173	Q7MU20	Uncharacterized protein PG_1752
0.010413	1.599755	Q7MX43	50S ribosomal protein rplM
0.001545	1.640403	Q7MTN8	30S ribosomal protein rpsK
0.03606	1.654393	Q7MUN0	Conjugative transposon protein TraK PG_1478
0.034886	1.741934	Q7MV71	Uncharacterized protein PG_1219
0.004164	1.759691	Q7MTM9	50S ribosomal protein L18 rplR
0.035604	1.762479	Q7MXB9	Uncharacterized protein PG_0286
0.001462	1.803937	Q7MWP8	Pyruvate-flavodoxin oxidoreductase PG_0548
0.025725	1.923307	Q7MUN3	Conjugative transposon protein TraN PG_1475
0.013816	2.000637	Q7MUN2	Conjugative transposon protein TraM PG_1476
0.033231	2.088482	Q7MT11	Peptidase, M23/M37 family PG_2192
0.010196	2.237788	Q7MTJ5	50S ribosomal protein L33 rpmG
0.000381	2.247797	Q7MWQ8	Aminoacyl-histidine dipeptidase pepD-2
1.46E-05	2.25966	Q7MW02	Uncharacterized protein PG_0866

## Discussion

We report, here, on the first RNA-binding protein (RBP) characterized in *P. gingivalis* that we designate RbpPg1. Although the oral pathogen *P. gingivalis* needs to rapidly adapt to ever-changing environmental conditions in the oral cavity and riboregulation allows for more fine tuning of protein levels and function, so far, not much is known regarding small RNAs as well as RNA-binding proteins in this organism (Hirano et al., 2012; Phillips et al., 2014). The RNA-binding protein we report on is biologically significant as it allows the organism to survive with host cells. The possible mechanisms involve modulation of metal homeostasis, protease activity, and ability to grow with low hemin levels.

The RbpPg1 is an RRM-1 protein. Interestingly, the RRM domain, also known as the ribonucleoprotein (RNP) domain, is the oldest RNA-binding domain reported and has been found widely in higher eukaryotes, but recent reports are emerging with updates on its presence in most domains of life, including bacteria and viruses (Maris et al., 2005; Cléry et al., 2008; Mozumdar and Roy, 2024). P*. gingivalis*, being an anaerobic organism, may have more in common with earlier ancestors carrying the first forms of RRM-1 as it is an anaerobic bacterium and, thus, evolved in the pre-oxygen area and possibly carries an earlier ancestor of the proteins found in eukaryotes (Hentze et al., 2018; Dolcemascolo et al., 2024; Mozumdar and Roy, 2024). A pointing fact is the existence of the RbpPg1 in the small form of just the RNA-binding domain, while in higher eukaryotes, it is usually fused to other domains, thus facilitating the co-localization of the catalytic domains with RNA (Takagaki and Manley, 1997; Xu et al., 2022).

RNA-binding proteins, so far, have been well characterized in other Gram-negative bacteria. *E. coli* and *Salmonella enterica* are paradigms for the Hfq RNA-binding protein that also serves as a chaperone for sRNAs (Vogel and Luisi, 2011; Wagner, 2013; Hoekzema et al., 2019; Lalaouna et al., 2021; Kavita et al., 2022). However, there is a major difference between the phyla *Proteobacteria* and *Bacteroidota (* Woese, 1987; von Mering et al., 2007). The latter separated from the eubacterial branch before other groups and thus are significantly different (Woese, 1987). The Bacteroidota group emerged early as a branch of distinct bacteria relying on anaerobic metabolism that further distinguishes them from the many aerobic organisms. Within the group are both oral and gut bacteria. The oral Bacteroidota, such as *P. gingivalis*, are asaccharolytic and rely on peptides for growth, while the gut ones are well known for their extensive carbohydrate utilization (Lamont and Jenkinson, 1998; Shi et al., 2007; Singh, 2019; Madej et al., 2020; Shin et al., 2024). With such stark nutritional requirements, different and unique regulatory mechanisms would also be expected. Recently, RBP was reported to regulate the ability of *B. thetaiotamicron* to grow with starch (Prezza et al., 2022; Rüttiger et al., 2025). It is noteworthy that the RBP required was also an RRM protein.

The N-terminal RRM-1 of CsfF-64 in *H. sapiens* was the closest protein structure hit to PG0627. This is a single ferredoxin-like fold RRM-1 motif that in RbpPg1 is followed by a disordered C-terminus. The disordered C-terminus is reminiscent of a similar structure at the C-terminus of the Hfq chaperone (Santiago-Frangos et al., 2016; Santiago-Frangos et al., 2017). Just as in the case of Hfq, CsrM, and ProQ that are found in a single copy on the genomes of Proteobacteria, we also found a single copy of the RbpPg1 on the genome of *P. gingivalis* strains. This is in contrast to the reports showing multiple copies of RRM-1 proteins in other Bacteroidota such as *B. thetaiotaomicron* and *B. fragilis (* Herren et al., 2003; Adams et al., 2021; Rüttiger et al., 2025). Although for RRM-1 motif proteins, the similarities of the multiple copies of the proteins vary vastly. Furthermore, the identity with the *P. gingivalis* RbpPg1 is limited, especially for the *B. thetaiotaomicron* RbpA, RbpB, and RbpC.

The CstF-64 was shown to bind a wide range of G-/U-rich sequences on single-stranded RNA with comparable affinity, yet was not highly specific for any one target sequence (Pérez Cañadillas and Varani, 2003). The *B. thetaiotaomicron* RbpB was shown to be more specific for a motif 1 (5′-GUAGGAUA-3′) binding it with a *KD* of 5.1 μM, while it did not bind a probe containing a 5′UCCUGUGC-3′ motif, thus demonstrating some degree of specificity (Adams et al., 2021). The affinity of RbpB was shown to be comparable to that of other RNA-binding proteins (Azam and Vanderpool, 2018; Adams et al., 2021; Hong et al., 2023; Harini et al., 2025). Recent studies have established the sequence enriched in RbpB CLIP studies and has shown that the binding is sequence specific and is in nanomolar range and, thus, an order of magnitude lower than that reported for Hfq and ProQ (Lease and Woodson, 2004; Fender et al., 2010; Smirnov et al., 2016). RBPs, such as Hfq, are known to bind to various motifs and ribosomal binding sites to instill their effect in degrading or stabilizing RNA, as well as inhibiting translation among other effects (Vogel and Luisi, 2011). In *B. thetaiotaomicron*, the RbpB was shown to interfere with FopS sRNA binding and de-repress glycan transporter levels (Rüttiger et al., 2025). This protein has 48% identity and 67% similarity over 83 aa and has not been identified as an ortholog of RbpPg1.

Although RNA-binding motif is well established, we still verified the RNA-binding capabilities of the RbpPg1 protein. Using EMSAs and RNA pulldowns followed by sequencing and identification of RNA fragments binding to RMN, we unequivocally demonstrate that the protein binds RNA. Our pulldown studies point to RbpPg1 binding to the PG2090 transcript coding for a putative zinc efflux pump. The same results were derived using two forms of RbpPg1 as follows: His tagged and HaLo tagged, and thus, we consider the investigation of the mechanisms of interaction between the transcript the RNA-binding protein as an attractive follow up direction. We did observe changes in the protein levels but not at the transcript level. This possibly could be due to the protein affecting the translation of the transcript but not its stability, and details of the mechanisms are yet to be established. We also used an RNA probe in our EMSA studies that carried the IRE recognition motif as well as other motifs found to bind RRM; however, more defined studies using RIP-Seq assay and CLIP (Smirnov et al., 2017; Andresen and Holmqvist, 2018; Hoekzema et al., 2019; Melamed, 2020) and verification of the binding are needed to better define the RbpPg1-binding sites. Such studies will also determine whether multi-molecule complexes are forming or sRNAs are involved in the interactions.

RbpPg1 plays a role in metal homeostasis. Its role may be explained by its ability to bind the zinc efflux pump gene, which may interfere with its function. Indeed, elevated levels of zinc are found in the RbpPg1 mutant strain when compared to that of the wild type. A zinc efflux protein playing a role in metal toxicity has been reported in *P. gingivalis (* Gains et al., 2023). Also, there are several efflux pumps encoded on the genome of the bacterium that are yet to be characterized. Indeed, the function, mechanism of action, and regulation of the protein encoded by PG2090 are yet to be determined. As zinc homeostasis is crucial for microbial survival in the presence of host cells, better understanding of its biological function and regulation is needed (Djoko et al., 2015).

In agreement with the putative role of RbpPg1, *P. gingivalis* mutants deficient in the protein were more sensitive to zinc. The molecular mechanisms of the regulation remain to be determined, and further studies are envisioned to be done in combination with the earlier reported Czc-efflux pump mutants to better define zinc homeostasis. Not only may zinc overaccumulation reduce the ability of the bacteria to grow, it may also do so via both direct and indirect mechanisms. Direct mechanisms are a replacement of other crucial metal co-factors (such as manganese), and the indirect mechanisms may involve generation of oxidative stress (Djoko et al., 2015; Harbison-Price et al., 2020).

We identified orthologs of PG2090 in *B. fragilis* and *T. forsythia* but not in *B. thetaiotaomicron* and *P. intermedia.* It is noteworthy that the presence of orthologs of RbpPg1 on genomes of Bacteroidota correlated with the presence of PG2090 orthologs indicating that this may be a regulatory mechanism not only confined to metal regulation in *P. gingivalis.* We found it remarkable that the oral pathogen *P. intermedia*, although also a member of Bacteroidota, did not code for either RbpPg1 and PG2090 orthologs. The possible explanation could be the difference in nutritional capability of the bacterium that can also utilize carbohydrates for growth.

Proteases are crucial virulence factors for *P. gingivalis* involved in array of functions ranging from nutrient provision to facilitating host pathogen interaction and ending on evading host responses (Potempa et al., 2003; Guo et al., 2010). The gingipains are especially significant due to the fact that 80% of the high proteolytic activity of *P. gingivalis* is due to the Arg-X and Lys-X gingipains (Kadowaki et al., 1998; Potempa et al., 1998; Curtis et al., 1999a; Lewis et al., 1999; Shi et al., 1999; Curtis et al., 2001; O-Brien-Simpson et al., 2003; Abe et al., 2004). We do show that the RbpPg1 is required for protease activity. Examination of our proteomics data shows that the levels of the RgpB protein are reduced compared with the parental strain. However, the levels of RgpA and Kgp were unaffected pointing out to alteration of post-translational modifications, such as glycosylation, that are necessary for a full activity of the proteases (Rangarajan et al., 1997; Aduse-Opoku et al., 1998; Curtis et al., 1999b). Indeed, three glycosyltransferases (PG0118, PG2223, and PG1135) as well as PorT and PorR, members of the secretion mechanism, had reduced protein levels (Shoji et al., 2002; Benedyk et al., 2019; Veith et al., 2022). The reduction in gingipain activity may also be due to the reduced protein level of the RgpB (Rangarajan et al., 2005).

Our studies show reduced survival of the RbpPg1 mutant with host cells when compared to the parental strain. We also demonstrate that the ability to associate with the host cells and be internalized by the non-phagocytic cells is not affected. Such reduced ability to survive with host cells could be due to the reduced ability to defend itself from reactive oxygen/nitrogen/sulfur species. Our study shows that RbpPg1 plays no role in response to peroxide stress; however, more comprehensive studies need to be done to better define the mutant sensitivity to an array of environmental stresses.

It is noteworthy that among the most upregulated proteins in the RbpPg1 mutant strain are mechanisms responsive to reactive radical challenge (oxidative, nitrosative, sulfur). Indeed, superoxide dismutase, ferritin, and thiol peroxidase are among the most upregulated in the mutant strain compared to those in the parental one suggesting that deletion of the RNA-binding activity predisposes the bacteria to radical stress. This appears in contrast with transcriptional data reported by the Fletcher group showing downregulation of PG0627 in the microarray and transcriptional screens in response to peroxide and nitric oxide exposure (Boutrin et al., 2012; McKenzie et al., 2012; McKenzie et al., 2015). Furthermore, the gene was not regulated in other transcriptional screens in response to nitrite and 6% oxygen (Lewis et al., 2009; Lewis et al., 2012; Yanamandra et al., 2012; Belvin et al., 2019). Many of the regulated proteins were previously observed to be regulated in the presence of 6% oxygen and by the ECF sigma factor encoded by PG1827 (Lewis et al., 2009) indicating a possible crosstalk between the regulatory mechanisms. The appearance of being under increased radical stress compounded by additional host immune defense stress makes the mutant strain very vulnerable in the oral cavity. We observed a correlation between the regulation of gene expression at both transcriptional and translational levels. However, not all differentially regulated proteins were also regulated at the transcript level indicating that RbpPg1 has multiple mechanisms of action. Detailed mechanisms as well as the *in vivo* binding capability of RbpPg1 will be surveyed in future studies using RIP-seq and CLIP (UV crosslinking and immunoprecipitation) approaches (Smirnov et al., 2017; Andresen and Holmqvist, 2018; Hoekzema et al., 2019; Melamed, 2020). The RNA derived from the RNA–protein complexes will be sequenced and unique reads mapped and quantified using controls as in other *in vitro* pulldowns.

## Data Availability

The RNAseq data have been deposited to the NCBI Gene Expression Omnibus (GEO) and available under the accession number GSE168570 (“Role of RNAbindingprotein, Pgr, in *P. gingivalis*”). The mass spectrometry proteomics data have been deposited to the ProteomeXchange Consortium via the PRIDE partnerrepository with the dataset identifier PXD034144 (“Role of RNA binding protein PgRBP1 in alteration of protein profile of *Porphyromonas gingivalis*”).

## Supplementary material

Supplementary Table 1 Plasmids and bacterial strains used in this study.

Supplementary Table 2 Primers used in this study.

Supplementary Table 3 Binding reaction 1 using IRE control RNA from The Light Shift Chemiluminescent RNA EMSA Kit.

Supplementary Table 4 Binding reaction 2. Dose-dependent binding reaction of RbpPg1 using IRE control RNA from The Light Shift Chemiluminescent RNA EMSA Kit.

Supplementary Table 5 Binding reaction 3 using RbpPg1 and synthesized biotinylated RNA probes.

Supplementary Table 6
*P. gingivalis* genes most significantly bound to His-tagged RBP compared to His-tagged HUβ determined using RNA-seq analysis. A. Locus ID based on Los Alamos, B. Definition based on BROP genome viewer, C. Reads per kilo base per million mapped reads (RPKM)-His-tag RbpPg1, D. Reads per kilo base per million mapped reads (RPKM)-His-tag HUβ, E. Ratio of the number of reads that align at each position from RBP to the reads from HUβ.

Supplementary Table 7
*P. gingivalis* genes most significantly bound to HaLo-tagged RbpPg1 (RBP) compared to HaLo-tagged HUβ determined using RNA-seq analysis. A. Locus ID based on Los Alamos, B. Definition based on BROP genome viewer, C. Reads per kilo base per million mapped reads (RPKM)-His-tag RbpPg1, D. Reads per kilo base per million mapped reads (RPKM)-His-tag HUβ, E. Ratio of the number of reads that align at each position from RbpPg1 to the reads from HUβ.

Supplementary Table 8 RNASeq analysis. WT vs. Mut. 4 biol replicates done in iron replete and iron deplete conditions.

Supplementary Table 9 RNAseq analysis in Mut vs. WT grown in iron deplete conditions.

Supplementary Table 10
*P. gingivalis* whole proteome quantification. All proteins identified.

Supplementary Table 11 Statistically significant regulated proteins mutant vs. WT.

Supplementary Table 12 Statistically significant regulated proteins complemented vs. WT.

Supplementary Table 13 Statistically significant regulated proteins mutant vs. complemented.

Supplementary Figure 1 RbpPg1 has characteristics of an RNA-binding protein. InterPro repository of databases for protein sequence analysis shows that RbpPg1 has nucleotide-binding domain (similar to RNA-binding domains of various ribonucleoproteins) and an RNA recognition motif (RRM) domain along residues 3–82.

Supplementary Figure 2 Intracellular iron content via ferrozine assay of W83 and V3139. Graph depicts three biological sets of triplicates. Optical densities were converted to concentrations using an iron standard curve with concentrations of 0.5, 1.0, 1.5, 2.0, and 2.5 ppm from 100-ppm stock solution.

Supplementary Figure 3 Role of RbpPg1 in *P. gingivalis* growth under hemin-replete and hemin-deplete conditions in the presence/absence of nitrosative stress and oxidative Stress. **(A–D)** Growth of *P. gingivalis* W83 (parental) and V3139 (RbpPg1 mutant) with nitrate or nitrite in heme-replete and heme-depleted conditions. **(E)** Disk diffusion assay of *P. gingivalis* WT and V3139 mutant grown with 1% or 5% hydrogen peroxide.

Supplementary Figure 4 RbpPg1 has no effect on attachment and internalization of *P. gingivalis* into non-phagocytic cells. Total interactions and invasion of *P. gingivalis* strains. Fluorescent tagging with FITC was used to quantify total extracellular interactions between the bacterial strains and HUVECs. Quenching of extracellular FITC by trypan blue was used to quantify total invasion between the bacterial strains and HUVECs.
