# Supplementary material for: The Porphyromonas gingivalis RNA-binding protein is required for growth in high levels of zinc and persistence with host cells
Source: Front Cell Infect Microbiol. 2025 May 15;15:1569544. doi: 10.3389/fcimb.2025.1569544 (PMC12119571; doi:10.3389/fcimb.2025.1569544)
Supplement: Supplementary Table 1 — Plasmids and bacterial strains used in this study. [file Table1.docx]

**Supplemental Table 1. Plasmids and bacterial strains used in this study.**

| **Bacterial Species** | **Strain** | **Plasmid** | **Description** | **Reference** |
| --- | --- | --- | --- | --- |
| *Porphyromonas gingivalis*  *Escherichia coli* | W83  V3129  V3236  One Shot Top10  Α-Select Silver  BL21 | None  None  pG108-0627  pCR2.1-TOPO  pET-30a  pET-30a  pFC20K | Parental Strain  PG0627-insertion mutant  V3129 containing plasmid with an intact copy of PG0627  Kanamycin resistance  Ampicillin resistance  His-Tag  Kan coding sequence  His-Tag  Kan coding sequence  HaLoTag T7 SP6  Flexi Vector  Kanamycin Resistance | Lewis and Macrina, 1999  Lewis et al; unpublished  Lewis et al; unpublished  Invitrogen  Bioline  Novagen  Promega |

| Primer name | 5’-3’ sequence | Description |
| --- | --- | --- |
| 1F  1R  F1 new  R1 new  2F  2R  3F  3R | **RbpPg1 primers:**  CGA GGC CAT GGC TAT GAG TAT GAA CAT CTA CGT AG  GCG TGC TCG AGT CAA TAG CGA TCT TCG TGT CGG  CTT CCA GGG ATC CCC AGA ATT CGA TCT TCT TCT GAA AGC CTG  GCG CAC TCG AGT TAC TCC AGC CTC GAC AAT CGG  **HU alpha primers:**  CGA GGC CAT GGC TAT GAA CAA GAC AGA TTT TAT TGC AG  GGT GCT CGA GTT ACT TAA GTT CCA AAG TAG AGC  **HU Beta** **primers:**  CGA GGC CAT GGC TAT GAC GAA AGC TGA CGT AGT GAA CG  GGT GCT CGA GTT AGT CTT GTT TCA TCT GAC TC  **EMSA Primers:**  **Biotinylated IRE Control RNA (125nM):**  5’ –UCCUGCUUCAACAGUGCUUGGACGGAAC—3’ –Biotin  **Unlabeled IRE Control RNA (10 μM):**  5’ –UCCUGCUUCAACAGUGCUUGGACGGAAC—3’  **Synthesized Biotin Labeled RNA Probes:**  **Long :**  5'- rGrGrG rGr**ArU rUrGrC rArC**rG rUrUrU rUrUrU rUrUrU rUr**GrC rArUrG**  rUrUrU rUrUrU rUrUrG rUrGrU rGrUrU r**ArUrU rGrCrA rC**rGrG rGrGrG rGrGrG  rG/3Bio/ -3'  **Short:**  5'- rUrUrG rGrUrU rUrUrA rUrUrG rCrUrU rUrGrC rArC/3Bio/ -3’ | RbpPg1F  RbpPg1R  RbpPg1F C-His  RbpPg1R C-His  HUalphaF  HUalphaR  HUbetaF  HUbetaR |

**Supplemental Table 2. Primers used in this study**

**Supplemental Table 3.** **Binding reaction 1 using IRE control RNA from The Light Shift Chemiluminescent RNA EMSA Kit.**

**
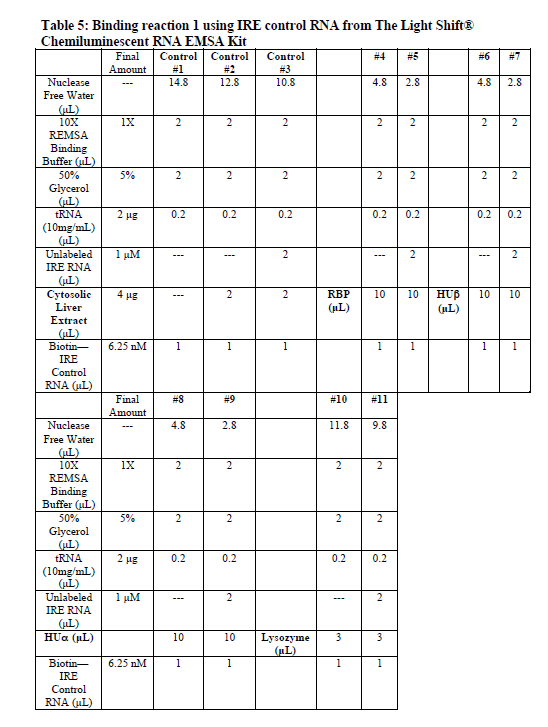
**

**Supplemental Table 4.** **Binding reaction 2. Dose-dependent binding reaction of RbpPg1 using IRE control RNA from The Light Shift Chemiluminescent RNA EMSA Kit.**

**
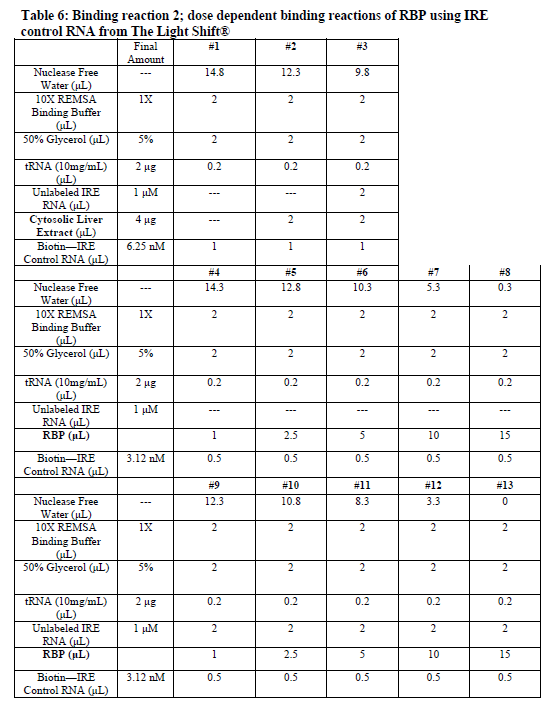
**

**Supplemental Table 5.** **Binding reaction 3 using RbpPg1 and synthesized biotinylated RNA probes**

**
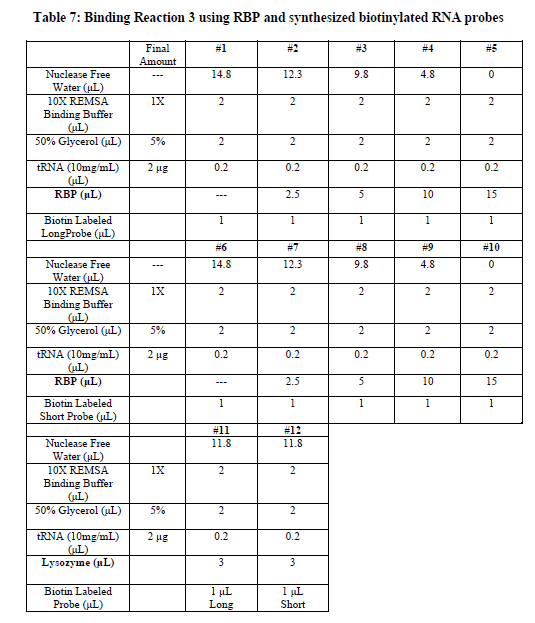
**

**Supplemental Table 6.** *P. gingivalis* genes most significantly bound to His-tagged RBP compared to His-tagged HUβ determined using RNA-seq analysis. A. Locus ID based on Los Alamos, B. Definition based on BROP genome viewer, C. Reads per kilo base per million mapped reads (RPKM)-His-tag RbpPg1, D. Reads per kilo base per million mapped reads (RPKM)-His-tag HUβ, E. Ratio of the number of reads that align at each position from RBP to the reads from HUβ.

**
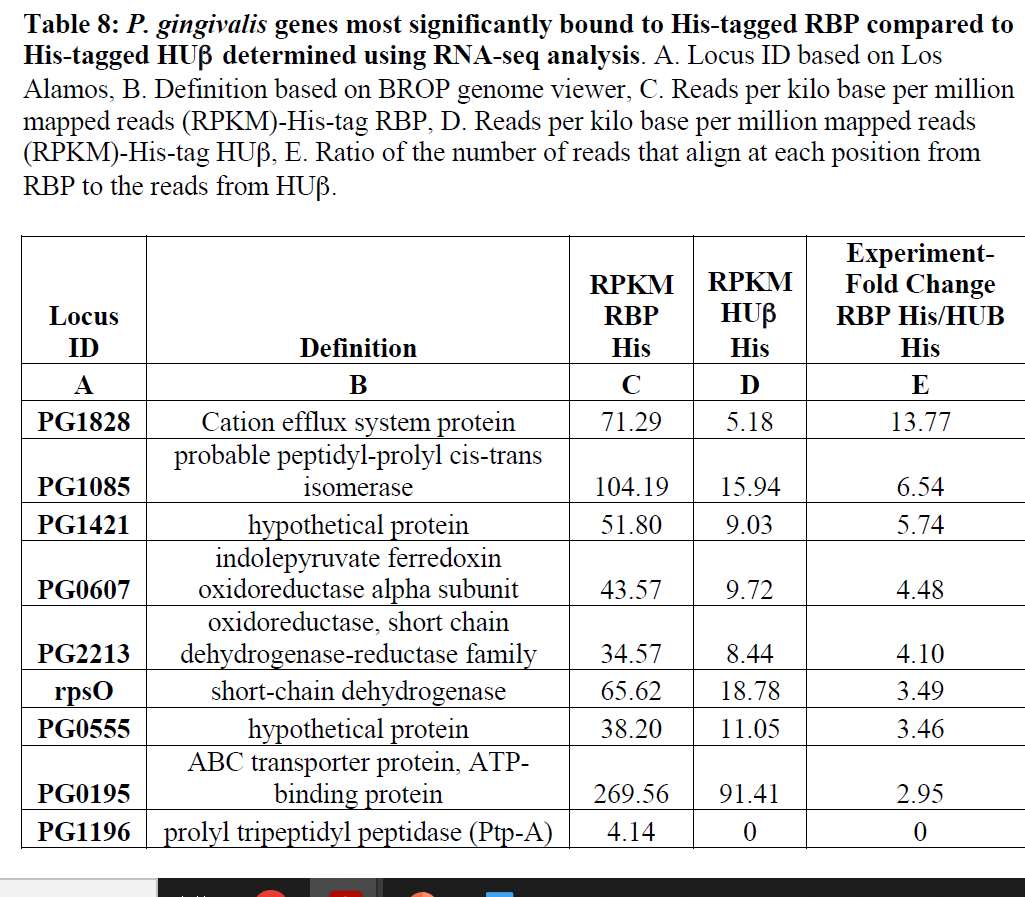
**

**Supplemental Table 7.** *P. gingivalis* genes most significantly bound to HaLo-tagged RbpPg1 (RBP) compared to HaLo-tagged HUβ determined using RNA-seq analysis. A. Locus ID based on Los Alamos, B. Definition based on BROP genome viewer, C. Reads per kilo base per million mapped reads (RPKM)-His-tag RbpPg1, D. Reads per kilo base per million mapped reads (RPKM)-His-tag HUβ, E. Ratio of the number of reads that align at each position from RbpPg1 to the reads from HUβ.

**
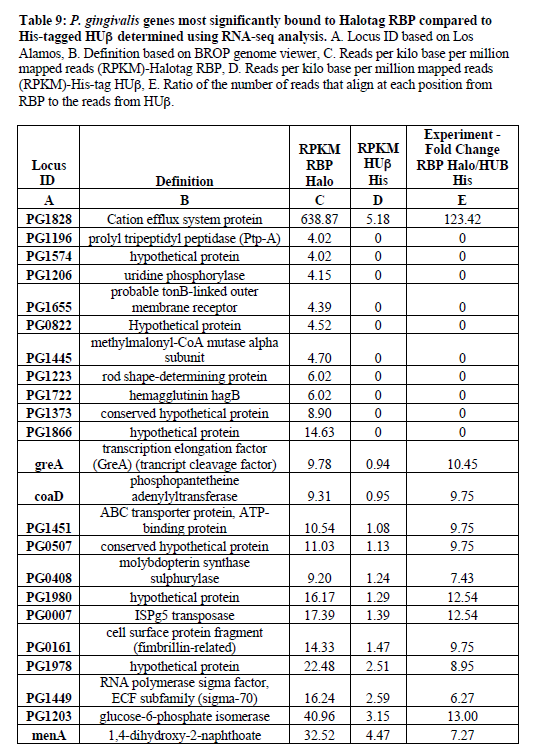
**

**
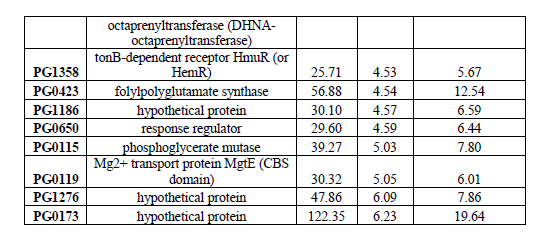

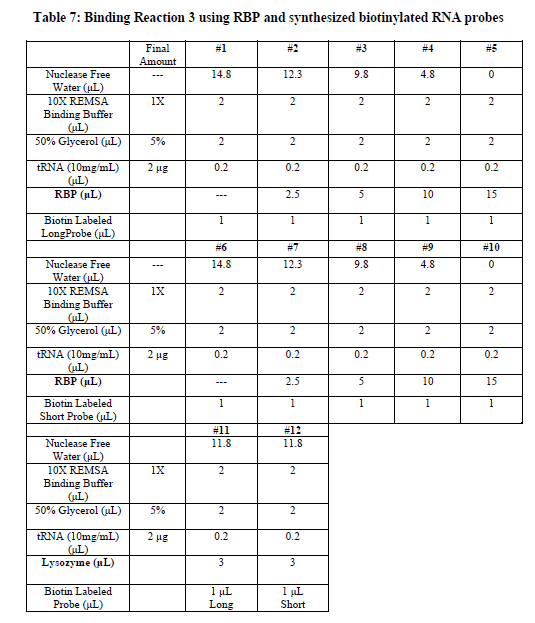
**
