# Supplementary material for: The Porphyromonas gingivalis RNA-binding protein is required for growth in high levels of zinc and persistence with host cells
Source: Front Cell Infect Microbiol. 2025 May 15;15:1569544. doi: 10.3389/fcimb.2025.1569544 (PMC12119571; doi:10.3389/fcimb.2025.1569544)
Supplement: Supplementary Figure 1 — RbpPg1 has characteristics of an RNA-binding protein. InterPro repository of databases for protein sequence analysis shows that RbpPg1 has nucleotide-binding domain (similar to RNA-binding domains of various ribonucleoproteins) and an RNA recognition motif (RRM) domain along residues 3–82. [file Presentation1.pptx]

## Slide 1
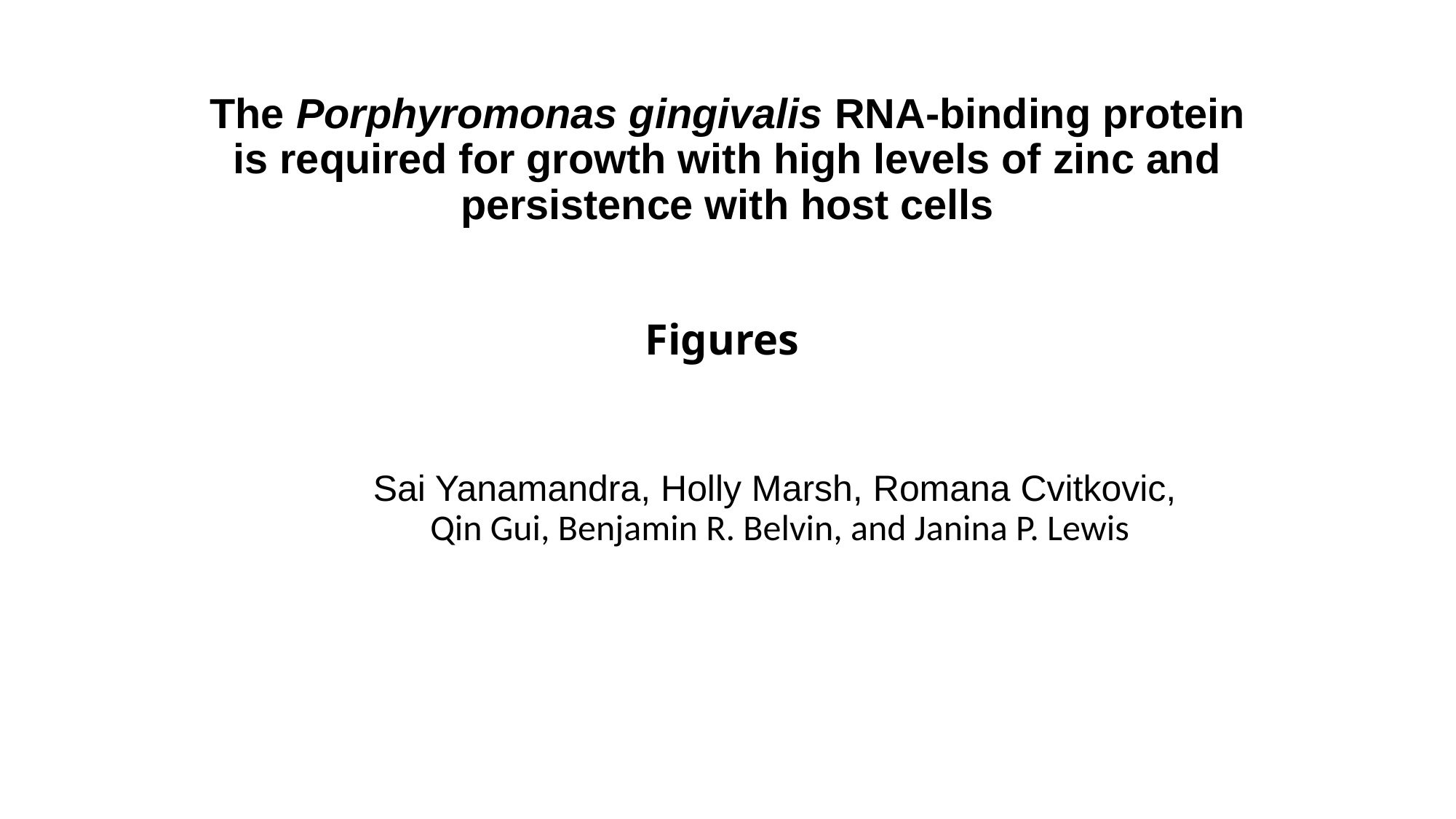

# The Porphyromonas gingivalis RNA-binding protein is required for growth with high levels of zinc and persistence with host cells Figures
Sai Yanamandra, Holly Marsh, Romana Cvitkovic, Qin Gui, Benjamin R. Belvin, and Janina P. Lewis

## Slide 2
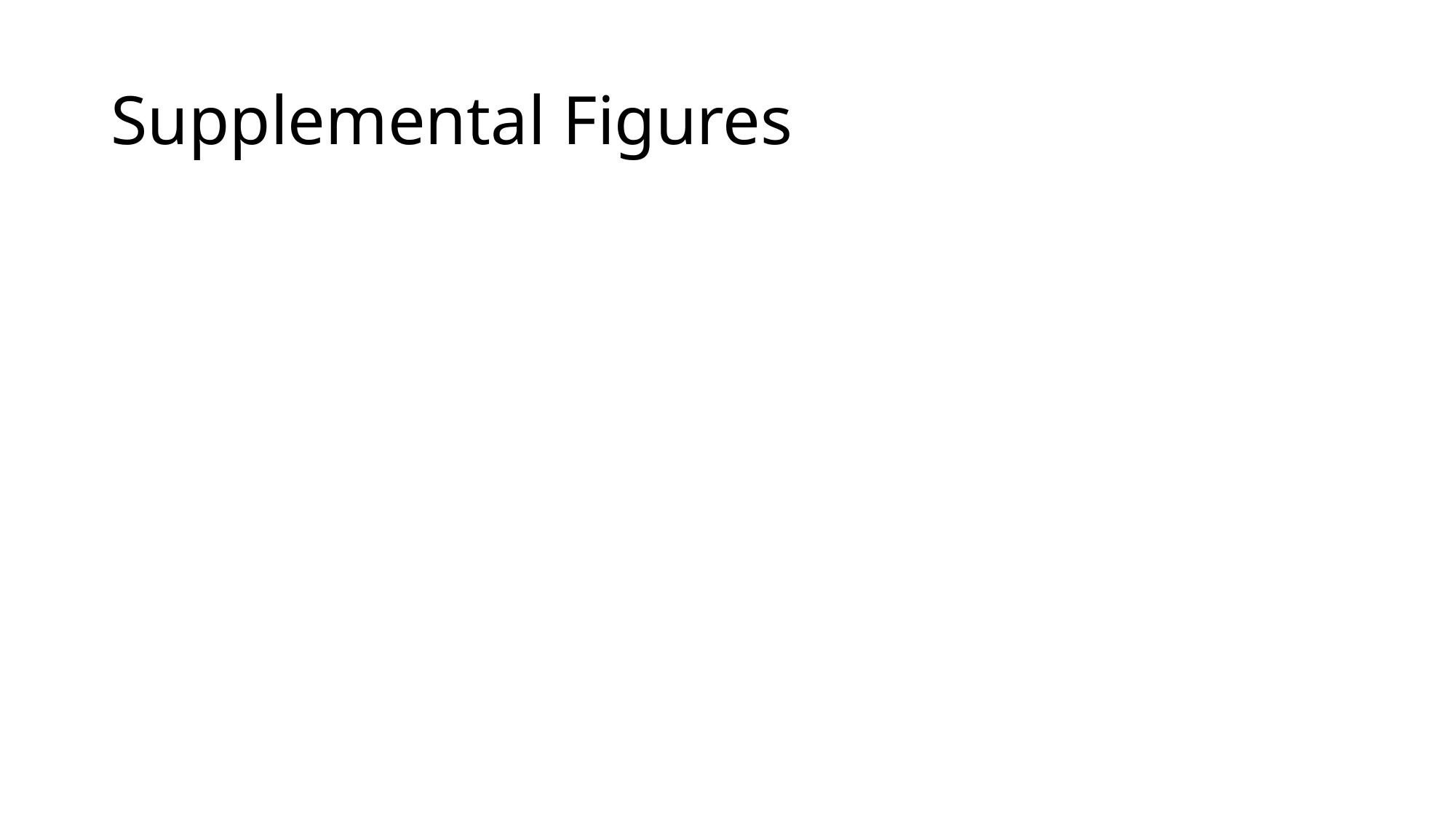

# Supplemental Figures

## Slide 3
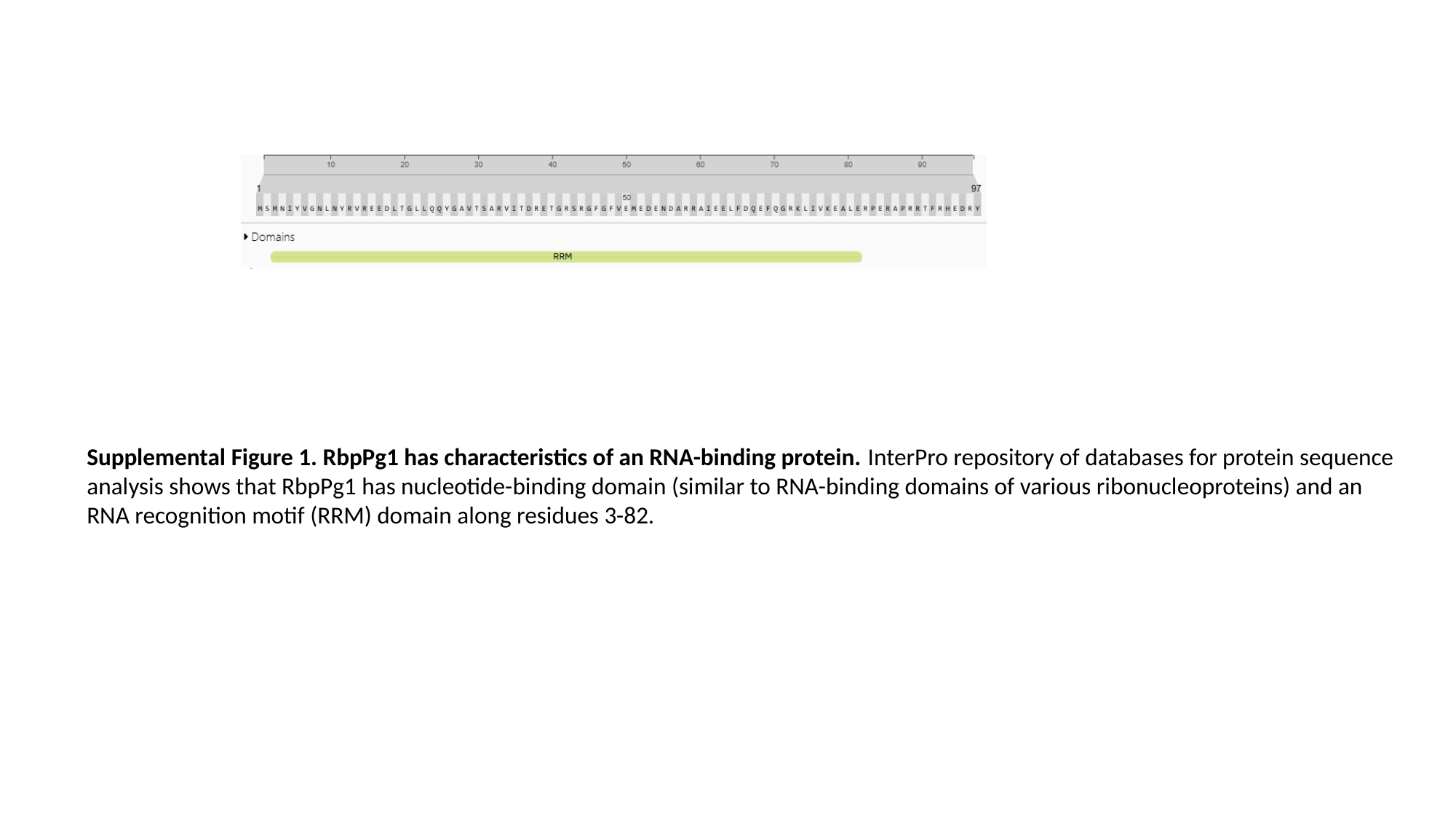

Supplemental Figure 1. RbpPg1 has characteristics of an RNA-binding protein. InterPro repository of databases for protein sequence analysis shows that RbpPg1 has nucleotide-binding domain (similar to RNA-binding domains of various ribonucleoproteins) and an RNA recognition motif (RRM) domain along residues 3-82.

## Slide 4
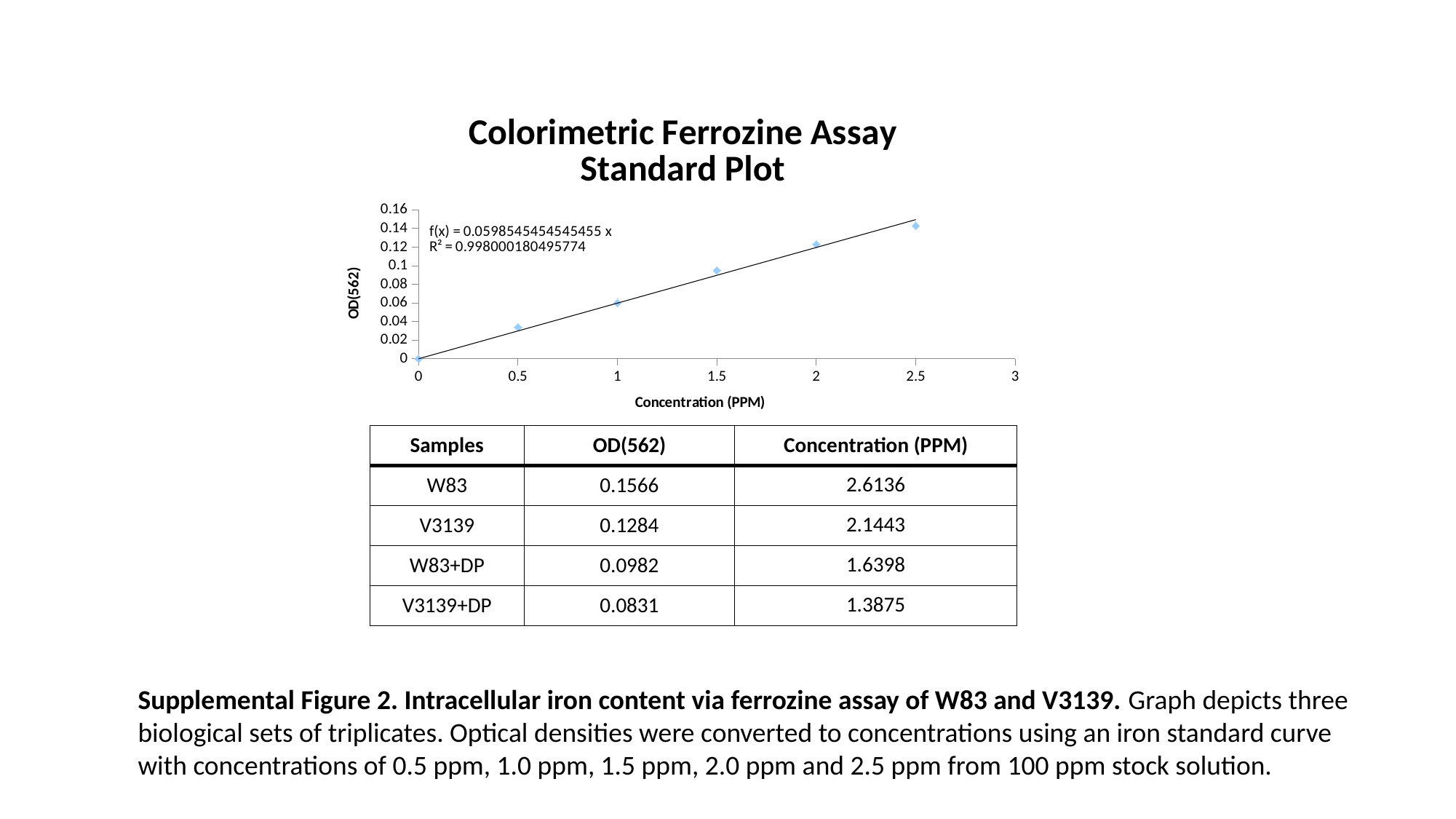

### Chart: Colorimetric Ferrozine Assay
Standard Plot
| Category | |
|---|---|| Samples | OD(562) | Concentration (PPM) |
| --- | --- | --- |
| W83 | 0.1566 | 2.6136 |
| V3139 | 0.1284 | 2.1443 |
| W83+DP | 0.0982 | 1.6398 |
| V3139+DP | 0.0831 | 1.3875 |
Supplemental Figure 2. Intracellular iron content via ferrozine assay of W83 and V3139. Graph depicts three biological sets of triplicates. Optical densities were converted to concentrations using an iron standard curve with concentrations of 0.5 ppm, 1.0 ppm, 1.5 ppm, 2.0 ppm and 2.5 ppm from 100 ppm stock solution.

## Slide 5
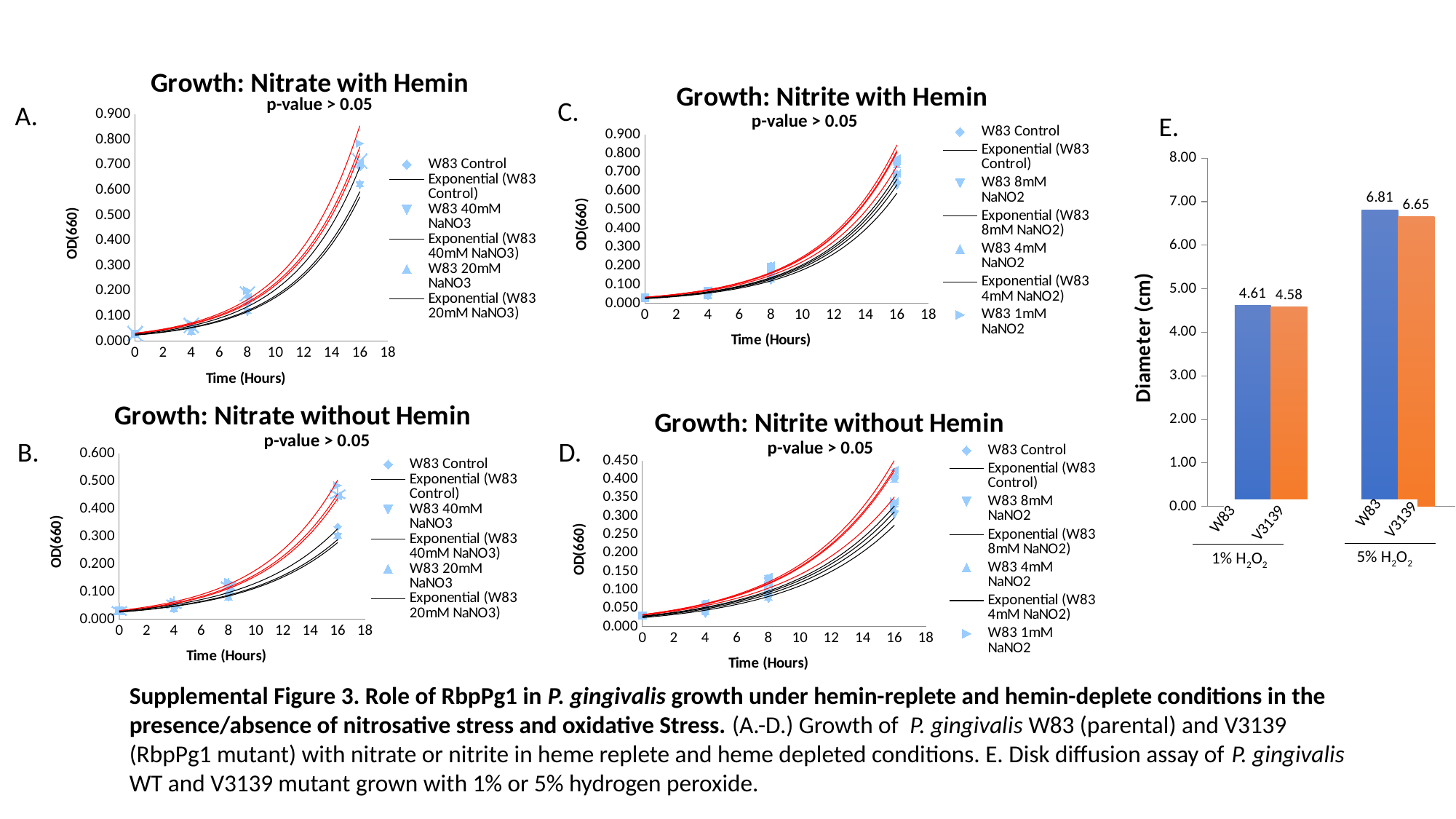

### Chart: Growth: Nitrate with Hemin
| Category | W83 Control | W83 40mM NaNO3 | W83 20mM NaNO3 | V3139 Control | V3139 40mM NaNO3 | V3139 20mM NaNO3 |
|---|---|---|---|---|---|---|
### Chart: Growth: Nitrite with Hemin
| Category | W83 Control | W83 8mM NaNO2 | W83 4mM NaNO2 | W83 1mM NaNO2 | V3139 Control | V3139 8mM NaNO2 | V3139 4mM NaNO2 | V3139 1mM NaNO2 |
|---|---|---|---|---|---|---|---|---|p-value > 0.05
C.
A.
p-value > 0.05
E.
### Chart
| Category | WT W83 | V3139 |
|---|---|---|
| 0.01 | 4.61 | 4.58 |
| 0.05 | 6.81 | 6.65 |
### Chart: Growth: Nitrate without Hemin
| Category | W83 Control | W83 40mM NaNO3 | W83 20mM NaNO3 | V3139 Control | V3139 40mM NaNO3 | V3139 20mM NaNO3 |
|---|---|---|---|---|---|---|
### Chart: Growth: Nitrite without Hemin
| Category | W83 Control | W83 8mM NaNO2 | W83 4mM NaNO2 | W83 1mM NaNO2 | V3139 Control | V3139 8mM NaNO2 | V3139 4mM NaNO2 | V3139 1mM NaNO2 |
|---|---|---|---|---|---|---|---|---|p-value > 0.05
D.
B.
p-value > 0.05
W83
W83
V3139
V3139
5% H2O2
1% H2O2
Supplemental Figure 3. Role of RbpPg1 in P. gingivalis growth under hemin-replete and hemin-deplete conditions in the presence/absence of nitrosative stress and oxidative Stress. (A.-D.) Growth of P. gingivalis W83 (parental) and V3139 (RbpPg1 mutant) with nitrate or nitrite in heme replete and heme depleted conditions. E. Disk diffusion assay of P. gingivalis WT and V3139 mutant grown with 1% or 5% hydrogen peroxide.

## Slide 6
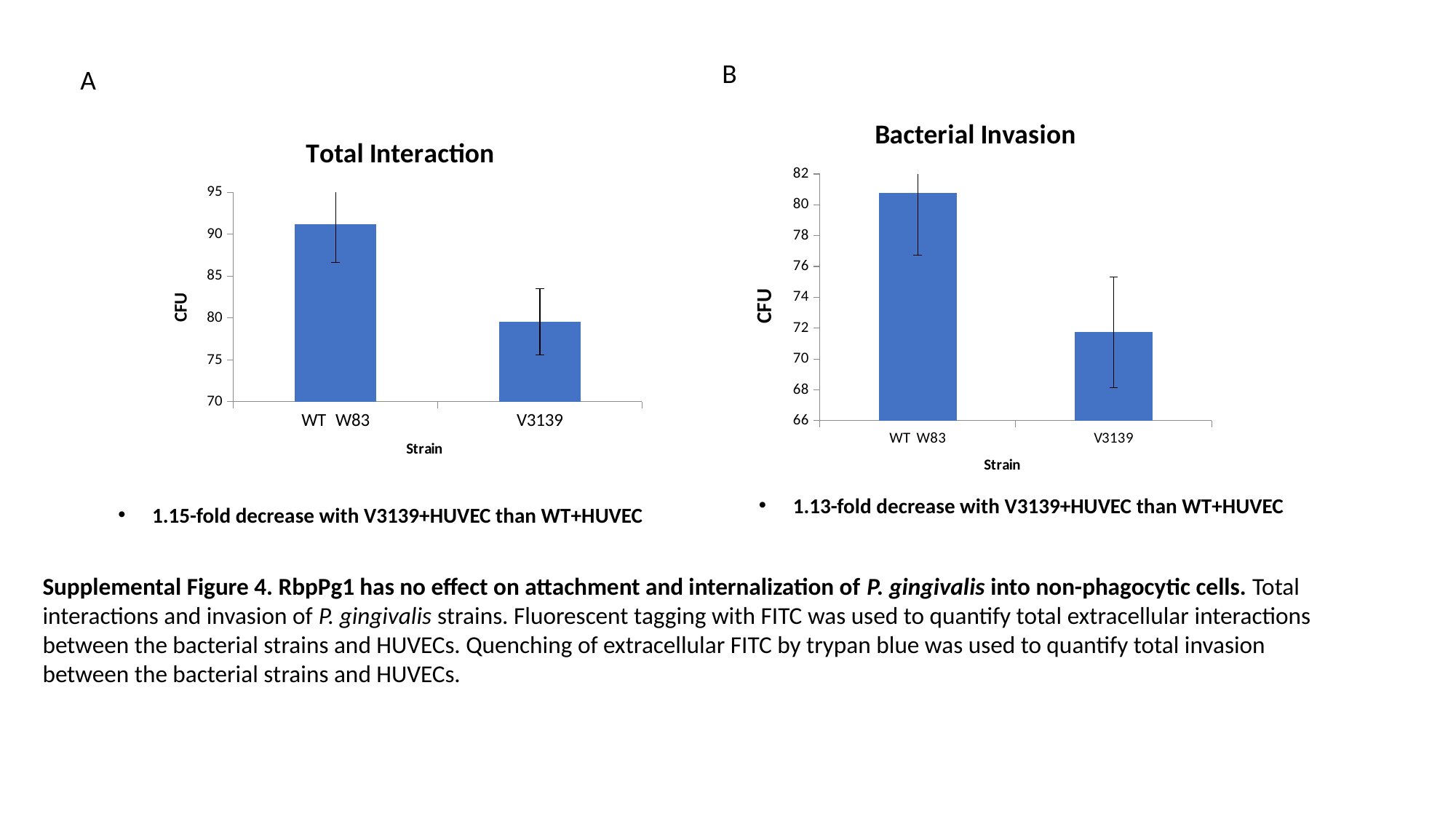

B
A
### Chart: Bacterial Invasion
| Category | |
|---|---|
| WT W83 | 80.752 |
| V3139 | 71.728 |
### Chart: Total Interaction
| Category | |
|---|---|
| WT W83 | 91.168 |
| V3139 | 79.552 |1.13-fold decrease with V3139+HUVEC than WT+HUVEC
1.15-fold decrease with V3139+HUVEC than WT+HUVEC
Supplemental Figure 4. RbpPg1 has no effect on attachment and internalization of P. gingivalis into non-phagocytic cells. Total interactions and invasion of P. gingivalis strains. Fluorescent tagging with FITC was used to quantify total extracellular interactions between the bacterial strains and HUVECs. Quenching of extracellular FITC by trypan blue was used to quantify total invasion between the bacterial strains and HUVECs.
